# Actinomycetota bioprospecting from ore-forming environments

**DOI:** 10.1099/mgen.0.001253

**Published:** 2024-05-14

**Authors:** César Aguilar, Amir Alwali, Madeline Mair, Lorena Rodriguez-Orduña, Haydeé Contreras-Peruyero, Ramya Modi, Carson Roberts, Nelly Sélem-Mojica, Cuauhtemoc Licona-Cassani, Elizabeth Ivy Parkinson

**Affiliations:** 1 Department of Chemistry, Purdue University, West Lafayette, IN, 47907, USA; 2 Tecnológico de Monterrey, Escuela de Ingeniería y Ciencias, Monterrey, Mexico; 3 Centro de Ciencias Matemáticas, UNAM, Morelia, Michoacán, Mexico; 4 Department of Medicinal Chemistry and Molecular Pharmacology, Purdue University, West Lafayette, IN, 47907, USA

**Keywords:** *Actinomycetota*, fluoride mines, genome mining, ore-forming environments, natural products

## Abstract

Natural products from *Actinomycetota* have served as inspiration for many clinically relevant therapeutics. Despite early triumphs in natural product discovery, the rate of unearthing new compounds has decreased, necessitating inventive approaches. One promising strategy is to explore environments where survival is challenging. These harsh environments are hypothesized to lead to bacteria developing chemical adaptations (e.g. natural products) to enable their survival. This investigation focuses on ore-forming environments, particularly fluoride mines, which typically have extreme pH, salinity and nutrient scarcity. Herein, we have utilized metagenomics, metabolomics and evolutionary genome mining to dissect the biodiversity and metabolism in these harsh environments. This work has unveiled the promising biosynthetic potential of these bacteria and has demonstrated their ability to produce bioactive secondary metabolites. This research constitutes a pioneering endeavour in bioprospection within fluoride mining regions, providing insights into uncharted microbial ecosystems and their previously unexplored natural products.

**Impact Statement**

This research has unveiled the unexplored biosynthetic potential of *Actinomycetota* in ore-forming environments. We have gained insights into the intricate interplay between bacterial metabolism and harsh environmental conditions. These findings will probably enable the discovery of new antibiotics and therapeutic leads from natural sources, helping to overcome the challenges of slowing natural product discovery rates. Moreover, it reveals the path for bioprospection in fluoride mining regions, significantly expanding our understanding of microbial ecosystems in these mines and their interesting natural product repertoire.

## Data Summary

- **Sequencing data**: All genome and metagenome sequences associated with this paper have been deposited at NCBI under the BioProject accession number PRJNA1007427.
- **Taxonomy assignment:** The scripts for the metagenome taxonomy assignments are available at Github: https://github.com/HaydeePeruyero/metagenomes_/tree/main
- **GNPS Networking:** All the mzxML employed for the GNPS Networking are accessible as a publicly available MassIVE data set identified by MSV000092746.

## Introduction

Historically, natural products (NPs) derived from bacteria have been crucial in developing medicinal and agricultural compounds [1]. Among the various bacterial phyla, *Actinomycetota* have emerged as a versatile source of antibiotics. They produce approximately two-thirds of naturally derived antibiotics and a diverse range of drugs with anticancer, antihelminthic, antifungal and immunosuppressive properties [2,3]. Despite early successes in NP discovery, scientists have encountered stagnation in identifying new compounds, with known molecules being frequently rediscovered. Genomic and bioinformatics data strongly indicate the potential for undiscovered NPs [4,6], but their identification requires intensified efforts.

NPs are hypothesized to be intricately linked to their environment [4,7,9]. *Actinomycetota* are widely distributed in different ecosystems, including soils, freshwater and marine environments, plants, and animals, highlighting their adaptability and ecological significance [10,14]. As a result, bacteria have developed numerous biochemical adaptations to face different stresses in their distinct ecosystems, resulting in a plethora of molecules with potential biotechnological applications [15,18]. Within their habitats, *Actinomycetota* play vital roles in nutrient cycling [19,20], defence mechanisms [14,21] and symbiotic interactions [22,23]. One practical approach to discovering novel NPs lies in exploring environments that have previously been underexplored. Under environmental stress, bacteria must develop chemical solutions to adapt to enhance their chances of survival in their particular environment [24]. We chose to focus on ore-forming environments because they are relatively underexplored and because of the challenges that these environments pose to survival: extreme pH, high salinity and poor nutrient availability [25]. In particular, fluoride mines and other fluorine-related soils present intriguing prospects for research due to often extreme abiotic conditions [10] and the limited ability of organisms in general to synthesize and metabolize fluorine-containing molecules [26]. Given the importance of fluorine-containing molecules in medicines [27], this is a particularly important topic. With no available information on the NP potential or bacterial diversity of these environments, we explored biodiversity and metabolism through metagenomics, metabolomics and evolutionary genome mining. Here we reveal the distinctive secondary metabolism present in these environments and demonstrate their capability to produce bioactive metabolites. To the best of our knowledge, this study represents the first analysis in bioprospection conducted in active areas associated with fluoride mining, paving the way for insights into unexplored microbial communities and their NP repertoire.

## Methods

### Soil sampling

The soil samples utilized in this study were collected from seven fluoride-related ore-forming mines in the USA. Three of the mines are located in Utah and the remaining four are in Illinois. The Utah soil samples were provided by J. D. Mallory, a geologist in the Fillmore Field Office of the Bureau of Land Management. The Illinois soil samples were provided by F. Brett Denny from the Illinois State Geological Survey (ISGS) at the Dunn-Richmond Center. The Illinois soil samples were obtained from the private land of Mr Lloyd Hogg, with his permission. All samples were collected from the Earth’s crust from depths not exceeding 10 m. The samples were collected using sterile plastic bags and promptly transported to the laboratory. Upon arrival, rocks and organic matter were removed from the soil via sifting. The soil was then stored at 4 °C to preserve the microbial community for subsequent analyses. The samples were collected during spring 2021.

### Mines in Utah

*Sample S1*. Coordinates: Topaz Mtn Rockhounding area, 039.696564° N 113.104368° W. Collected within the precincts of the Bureau of Land Management’s designated public rockhounding site. It was in a shady spot on the east-facing side of the mountain, which probably receives some morning sun. Tiny clear topaz crystals were noticed while collecting the sample. The sample was pulled from the inside of the vug. Weather: sunny, 21.1°C, windy. *Sample S3*. Coordinates: Bell Hill Mine, 039.699668° N 113.176308° W. This location is on an active claim, so we obtained an old sample. The date of sampling is unknown. *Sample S4*. Coordinates: Yellow Chief Mine, 39.7418634° N 113.1501906° W. Historically, this location was mined for uranium. The sandstone-type rock was mined for its ore. The area is rich in bentonite conglomerate. This spot gets a large amount of sunlight. Weather: sunny, 23.9 °C, windy.

### Mines in Illinois

*Spire Pile*: Coordinates: 37.521775° N 88.405497° W. Clay material sample clinging to fluorspar ore from pieces of raw ore before being run through the log washer. *Hicks Creek PMT Mine*: Coordinates: 37.522316° N 88.425298° W. A sample of clay soil material was taken from the PMT mining trench from which fluorite was extracted along the Pierce vein. *Lloyd Hogg Cut (near T and M and Slapout Mine)*: Coordinates: 37.519491° N 88.424126° W. The sample was collected from clay soil part of a weathered vein. People mine this vein intermittently for gravel spar or fluorite. *Minerva*. Coordinates: 37.543464° N 88.156335° W. The sample was taken from mine tailings

### Elemental analysis

Chloride, sulphate and nitrate concentrations were tested using the SW-846 Test Method 9056, which determines inorganic anions by ion chromatography [28]. Fluoride concentrations were tested using the methodology described in ASA #9 Part 3 Chapter 31 [29]. All the measurements were performed by the Alfa Chemistry testing lab company.

### Sequencing

Genomic DNA from isolated bacteria and DNA for metagenomic analysis were extracted using the 2.4.3 protocol ‘Preparation of Genomic DNA from Bacteria’ from the Current Protocols of Molecular Biology manual [30] with minor adjustments. Briefly, 125 ml flasks containing 25 ml of ISP-2 liquid medium were inoculated with 1 ml of pre-grown bacterial cells and incubated for 3–15 days at 30 °C and 120 r.p.m. The biomass was transferred into a sterile porcelain mortar for liquid nitrogen lysis. The crushed mycelium was mixed with 5 ml TE buffer supplemented with lysozyme (20 mg ml^−1^; Sigma-Aldrich), 25 % (w/v) SDS and proteinase K (20 mg ml^−1^; Fisher-Scientific), for chemical rupture. The lysate was cooled and extracted twice with equal volumes of chloroform/isoamyl alcohol (24 : 1, v/v). The aqueous phase was transferred for DNA precipitation with 2-propanol at 4 °C for 30 min. The pellet was obtained by centrifuging in an Eppendorf Centrifuge 5810 at 3200 ***g*** for 10 min. Genomic DNA (gDNA) was resuspended in deionized sterile water and checked for quality using absorbance 260 nm/280 nm and 230 nm /260 nm ratios and via electrophoresis using a 2 % agarose gel. For the isolation of environmental (eDNA), the same protocol was followed, but with 5 g of soil and without the use of liquid nitrogen for lysis.

### gDNA sequencing and assembly

The sample libraries were prepared with the Illumina DNA Prep kit. Whole-genome sequencing was performed on the Illumina NextSeq platform obtaining 2×151 bp. Before *de novo* assembly, read quality was assessed using FastQC v0.11.9 [31]. Any low-quality reads were filtered out. Adapters were removed and reads were trimmed based on quality to ensure only high-quality data were kept. Finally, the reads were filtered based on their k-mer coverage and normalized further to ensure a balanced representation.

### Hybrid assemblies

Hybrid approaches were adopted for enhanced assembly by combining Illumina reads with long-read Oxford Nanopore Technologies. To generate long reads, libraries were prepared using the rapid sequencing kit V14 (catalogue number SQK-RAD114), following the manufacturer’s instructions. These were sequenced on a Nanopore MinION device using a 24 h sequencing cycle and default parameters in the MinKNOW GUI 5.3.6 platform. The resulting long-read raw files and Illumina reads underwent *de novo* assembly using the Unicycler v0.4.8 assembler through the PATRIC genome assembly service [32,33]. The resulting assembled genomes were used for annotation, phylogenomic analysis and genome mining studies.

### eDNA sequencing

For metagenome sequencing, three independent DNA isolations were performed for each sample to mitigate potential DNA extraction biases, and the resulting DNA extracts were pooled together. Metagenomic sequencing was performed on the Illumina NextSeq platform obtaining 2×151 bp. Quality at each base in all the reads for both samples was verified by using FastQC [31]. Low quality nucleotides in pair ended reads were removed were removed with Trimmomatic (v0.38) [34], SLIDINGWINDOW:4 : 20 MINLEN:35 LEADING:20 TRAILING:20.

### Taxonomy assignment

Kraken 2.1.2 was used to trim genomes [35]. The taxonomy was determined using the biom file and the library phyloseq in R. The biom file was constructed with kraken-biom v1.2.0.

### Functional and taxonomic profiles for microbiome metabolic characterization

Gene prediction from metagenomes was conducted directly on the reads. The paired-end R1 and R2 files of each metagenome were subjected to quality assessment using Fastp v0.23.4 [36] with default parameters, except: length (40), trim_front 1 and 2 (5), trim_tail 1 and 2 (5), cut right and front mean quality (25). Subsequently, gene prediction was performed with FragGeneScan software v1.3.0 [37] using the illumina train dataset and default parameters for non-assembled metagenomes. For the functional annotation, we employed eggNOG v5.0, which operates with the KEGG Ortholog DB and uses internal default settings [38]. A functional orthologue is a term used in KEGG molecular networks, such as KEGG pathway maps, BRITE hierarchies and KEGG modules. In these networks, each node is assigned a KO identifier or K number which represents a functional orthologue. This identifier is defined manually based on experimentally characterized genes and proteins in specific organisms. These functional orthologues are then used to identify orthologous genes in other organisms based on sequence similarity. The level of ‘function’ may differ based on the context and the resulting KO grouping may correspond to a group of highly similar sequences in a limited organism group or a more divergent group [39]. In parallel, the output from fastP was utilized to conduct taxonomic assignments using Kraken v2.1.2 [35] with default parameters for paired-end reads. Abundance and counts at the species level were calculated using Bracken 2.8 [40] with default parameters, except: readLength (130), minReadLength (40) and threshold (1). For each taxon, counts per million were calculated from the eggnog output for each KO to generate a final matrix of taxon–KO abundance per sample. Sample normalization was achieved based on the mapped read count against the local gene catalogue to account for sequencing depth variations. Raw data of the analysis are provided in File S1, available in the online version of this article (*Supplementary_Files S1_Metag_R1_rawCounts_KOs* and *Supplementary_Files S1_Metag_R2_rawCounts_KOs* tabs).

### Calculation and drawing of Venn diagrams

Metagenomic data were categorized into *Actinomycetota* orthologues and bacterial, non-*Actynomycetota* orthologues. Orthologues belonging to other organisms were excluded. The unique KOs were manually filtered from the four datasets. Venn diagrams were created using https://bioinformatics.psb.ugent.be/webtools/Venn/ with default Symmetric/Colored parameters. The graphical output was obtained in SVG format. The .txt output file with the KO arrangement is available in File S1 (*Supplementary_Files S2_Venn_Diagram_txt* tab).

### Bacterial isolation

Each soil sample was sifted through a brass sieve with a 2 mm aperture size. The sieved soil was dried at 65 °C for 24 h to determine its dry weight. One gram of soil from each sample was resuspended in 10 ml of sterile Milli-Q Ultrapure water and the suspension was thoroughly mixed for 5 min. Subsequently, each suspension underwent serial dilution ranging from 10^−1^ to 10^−6^. Samples (500 µl) were then spread on four actinomycetotal nutrient agar plates using glass beads. *Streptomyces Isolation Media*: 0.4 g casein, 1 g starch, 0.5 g KNO_3_, 0.2 g K_2_HPO_4_, 0.1 g MgSO_4_, 0.1 g CaCO_3_, 15 g agar, 1 litre distilled water. *Humic Acid Media*: 1 g humic acid, 1.7 g KCl, 0.5 g Na_2_HPO_4_, 0.5 g MgSO_4_, 0.02 g CaCO_3_, 0.01 g FeSO_4_, 1 ml VB stock solution (added after autoclaving), 10 g agar, 1 litre distilled water. The VB stock solution contains the following: 50 mg VB1 (thiamine), VB2 (riboflavin), VB3 (niacin), VB6 (pyridoxine), B5 (d-calcium pantothenate), inositol and PABA (para-amino acid), and 25 mg B7 (biotin) dissolved in 100 ml distilled water. The VB stock solution is sterile filtered prior to addition to the media. *Starch nitrate*: 20 g starch, 1 g K_2_HPO_4_, 2 g KNO_3_, 0.5 g MgSO_4_, 3 g CaCO_3_, 2 g NaCl, 10 g agar, 1 ml of trace salt solution and 1 litre distilled water. *Starch casein*: 10 g starch, 0.3 g casein, 2 g KNO_3_, 0.05 g MgSO_4_, 2 g K_2_HPO_4_, 2 g NaCl, 0.02 g CaCO_3_, 0.01 g FeSO_4_, 10 g agar and 1 litre distilled water. *Trace salt solution*: 40 mg ZnCl_2_, 200 mg FeCl_3_.6H_2_O, 10 mg CuCl_2_.2H_2_O, 10 mg MnCl_2_, 10 mg Na_2_B_4_O_7_.10H_2_O, 10 mg (NH_4_)_6_Mo_7_O_24_.4H_2_O and 1 litre distilled water. Plates were incubated at 30 °C for 5 to 15 days. The most prominent colonies were isolated by subsequent streaking. Strains were maintained at −80 °C as glycerol stocks for further studies.

### Identification of bacteria by 16S rRNA gene sequencing

From all the isolated bacteria (Table S1), the 16S rRNA gene was amplified in three separate PCRs from gDNA using 0.5 µM primers [41], 0.2 mM of dNTP mix (Biolabs), 1× PCR buffer (NH_4_)_2_SO_4_, 1.5 mM MgCl_2_, 5 % DMSO and 1.0 U Taq polymerase (Thermo) final concentrations. Amplification conditions were as follows: 2 min at 95 °C to denature the DNA, followed by 35 cycles of denaturation at 95 °C for 30 s, primer annealing at 50 °C for 30 s and strand extension at 72 °C for 1.5 min on a BioRad thermal cycler. Primers and excess nucleotides were removed from the amplified DNA using a PCR clean-up kit (GeneJet). PCR products were submitted to electrophoresis on a 1 % agarose gel and DNA bands were visualized with SybrGreen Safe (Thermo) to confirm the 1.5–1.6 kb 16S rRNA gene size. The resulting clean DNA was quantified using a Qubit. Paired Sanger sequencing for all the 16S rRNA genes was performed by Genewiz using 40 ng of DNA with 25 pM of each primer. After removing ambiguous and low-quality bases, R1 and R2 files of each sample were manually assembled, and taxonomy assignment was performed by blasting against the bacterial NCBI database.

### Metabolomics

#### Isolation and growth media

The isolated strains were cultivated individually on five distinct solid media. *R4*: 0.5 % glucose, 0.1 % yeast extract, 0.5 % MgCl_2_.6H_2_O, 0.2 % CaCl_2_.2H_2_O, 0.15 % proline, 0.118 % valine, 0.28 % TES, 50 mg l^−1^ casamino acid, 100 mg l^−1^ K_2_SO_4_, 1× trace element solution, 12 g agar and 1 litre distilled water. *SFM*: 20 g mannitol, 20 g soy flour, 20 g agar and 1 litre tap water. *GYM*: 4 g glucose, 4 g yeast extract, 10 g malt extract, 2 g CaCO_3_, 12 g agar and 1 litre distilled water, adjusted to pH to 7.2 before adding agar. *ISP2*:10 g malt extract, 4 g yeast extract, 4 g glucose, 12 g agar and 1 litre distilled water, pH 7.2. *ISP4*: 10 g soluble starch, 1 g K_2_HPO_4_, 1 g MgSO_4_.7H_2_O, 1 g NaCl, 2 g ammonium sulphate, 2 g calcium carbonate, 1 mg FeSO_4_.7H_2_O, 1 mg ZnSO_4_.7H_2_O, 1 mg MnCl_2_.2H_2_O, 12 g agar and 1 litre distilled water, pH 7.2.

#### Metabolite extraction

Following 7 days of growth, the excreted metabolites from each culture were retrieved by freezing the agar followed by thawing and compressing the agar medium to release the liquid content (~15–20 ml per standard Petri dish), as has been previously described [42,43]. The culture broth from each strain cultivated in the five media was pooled into a single composite sample. Subsequently, each sample underwent centrifugation in an Eppendorf Centrifuge 5810 at 3200 ***g*** for 10 min, followed by filtration through 6.5-inch non-gauze milk filters to remove residual agar. A 95 % acetonitrile (ACN) extraction was then performed using HLB Waters 6 ml Vac Cartridge columns (200 mg sorbent, 30 µm). The solvent was removed via lyophilization, and the extracted metabolites were subsequently reconstituted in a 5 % DMSO solution.

For LC-MS separations were performed on an Agilent 1290 system, with a mobile phase flow rate of 0.40 ml min^−1^. The metabolites were assayed using a Waters BEH C18 column (1.7 µm, 2.1×100 mm), where the mobile phases were A (0.1 % formic acid in ddH_2_O) and B (0.1 % formic acid in ACN). Initial conditions were 95 : 5 A:B, held for 1 min, followed by a linear gradient to 20 : 80 at 26 min, then 5 : 95 at 28 min. Column re-equilibration was performed by returning to 95 : 5 A:B at 29 min and holding until 35 min. Mass analysis was done in positive ionization mode using an Agilent 6545 Q-TOF mass spectrometer with ESI capillary voltage of +3.5 kV. MS/MS was performed in Data Dependent Analysis (DDA) mode, with a range of 50–1700 *m*/*z*. Mass accuracy was improved by infusing Agilent Reference Mass Correction Solution (G1969-85001). Peak deconvolution, integration and statistical analysis were performed using MS-DIAL (v4.9.221218) [44]. Peak annotations were performed using the MassBank of North America MS/MS library, based on authentic standards (v16) (http://prime.psc.riken.jp/compms/msdial/main.html#MSP). Mass tolerances for identification were 0.005 Da for MS1 and 0.01 Da for MS2.

### GNPS workflow for molecular networks

#### MS data processing and analysis

Initial data acquired from the Agilent 6545 Q-TOF system in .d format were converted with MSConvert 3.0 [45] into .mzXML format under a 32 bit format without zlib compression, including data smoothing, baseline correction, peak picking with Vendor and 1–2 MS-Levels. The converted files were submitted to the Global Natural Product Social Molecular Networking (GNPS) database and data analysis server [46,47].

#### GNPS classical molecular networking

Molecular networks were generated on the GNPS platform using specific parameters, including a mass tolerance of 0.03 Da, a cosine score of 0.65, a minimum of 4 matched fragment ions and a minimum cluster size of 3 spectra. For automated chemical classification, the MolNetEnhancer tool [48] was employed. Library searches applied similar criteria with a cosine score of 0.5 and a minimum of 4 matched peaks. The GNPS libraries used for the comparison are listed in Table S2. A list of library hits is provided in File S1 (*Supplementary_Files S3_MOLNETENHA-superclass-attri* tab), adhering to the metabolomics standards initiative [49]. We used Graphia v4.2 software to generate visual network representations [50].

#### Structural similarity network annotation

We submitted the Classic GNPS-MN output file to the Structural Similarity Network Annotation Platform to predict the identities of compound clusters based on MS features (SNAP-MS) [51]. The NP-Bacterial Atlas library and default parameters were employed.

### Genome mining

The six strains described herein underwent evolutionary genome mining analysis following the latest protocol of Cheverette *et al*. [52]. Briefly, we used antiSMASH 7.0 [53] in the ‘relaxed’ strictness setting to identify specialized metabolite gene clusters. A total of 175 predicted biosynthetic gene clusters (BGCs, in .gbk file format) were then subjected to local processing using the BiG-SCAPE v1.1.5 software [54], with reference to the MiBIG 3.0 database [55] (accessed 20 June 2023). The BiG-SCAPE analysis incorporated the Pfam 35.0 database version. To ensure comprehensive results, the singleton parameter was chosen within BiG-SCAPE, encompassing BGCs with distances of 0.3 as the cut-off value for BGCs and 0.75 for gene cluster families (GCFs). Additionally, the hybrids-off parameter was activated to prevent redundancy among hybrid BGCs. The Cytoscape v3.10.1 software was used to visualize the networks [56].

#### BiNI

To assess the novelty of the biosynthetic potential encoded by an isolate, we employed a metric that primarily considers the number of clusters (*n*) identified by antiSMASH, along with the distance (*d*) sampling test result obtained from the BiG-FAM platform (threshold value >900). This metric is represented by the equation BiNI=∑*d*/*n* [57].

### Phylogenomic classification

The genome sequences were submitted to the Type (Strain) Genome Server (TYGS) at https://tygs.dsmz.de, to facilitate a comprehensive taxonomic analysis based on whole-genome data [58]. Briefly, nomenclature, synonymy and related taxonomic literature were sourced from TYGS’s affiliated database, the List of Prokaryotic Names with Standing in Nomenclature (LPSN), accessible at https://lpsn.dsmz.de [59]. The results were retrieved from TYGS on 3 June 2023.

#### Determination of closely related type strains

Employing the MASH algorithm [60], all genomes were matched with the type strains in the TYGS database. For each genome, ten type strains with the lowest MASH distances were selected. A supplementary analysis by extracting 16S rDNA gene sequences using RNAmmer was conducted [61]. These sequences were then BLASTed [62] against the 16S rDNA gene sequences of the 19 084 type strains available in the TYGS database. Fifty matching type strains for each genome based on bitscore were selected. Distances were calculated further using the Genome blast Distance Phylogeny (GBDP) method with the 'coverage' algorithm and distance formula d5 [63]. These distances guided the identification of the ten closest type strain genomes for each genome.

#### Pairwise comparison of genome sequences

For phylogenomic relationships, pairwise comparisons among the dataset genomes using the GBDP method were conducted. Intergenomic distances were accurately determined using the 'trimming' algorithm and distance formula d5 [63]. Digital DNA–DNA hybridization (dDDH) values and confidence intervals were calculated using the GGDC 3.0 tool’s recommended settings [59,63].

#### Phylogenetic inference

Using the intergenomic distances, a balanced minimum evolution tree with branch support using FASTME 2.1.6.1 was built [64]. Each branch’s strength was evaluated through 100 pseudo-bootstrap replicates. The trees were rooted at the midpoint and visualized with PhyD3 [65].

#### Type-based species and subspecies clustering

Species clustering centred on type strains was conducted using an established method with a 70 % dDDH radius around the 19 chosen type strains, as outlined earlier. For subspecies-level clustering, a dDDH threshold of 79 % was applied, effectively revealing meaningful subspecies groups within the dataset.

### Bioactivity testing

#### Antibiotic activity

Pathogens tested included *Escherichia coli* ATCC 25922, *Escherichia coli* BAA2469, *Staphylococcus aureus* ATCC 29213, *Staphylococcus aureus* NRS3, *Klebsiella pneumoniae* ATCC 27736, *Klebsiella pneumoniae* BAA2146, *Acinetobacter baumannii* ATCC 19606, *Acinetobacter baumannii* KB349, *Pseudomonas aeruginosa* PAO1, *Pseudomonas aeruginosa* PA1000, *Enterococcus faecalis* ATCC 19433, *Enterococcus faecium* S235 and *Bacillus subtilis* 6633. All strains except *B. subtilis* 6633 were obtained from P. J. Hergenrother (UIUC). *B. subtilis* 6633 was obtained from W. W. Metcalf (UIUC). *Escherichia coli, K. pneumoniae, A. baumannii* and *P. aeruginosa* were maintained on Mueller Hinton Broth 2 (Sigma-Aldrich). *S. aureus, Enterococcus* species and *B. subtilis* were maintained on Bacto Brain Heart Infusion. Bioactivity testing was performed using the CLSI microbroth dilution assay protocol, with extracts being examined at 100 µg ml^−1^ (2 % DMSO final). All wells were compared to a vehicle control. A positive control (64 µg ml^−1^ ciprofloxacin) was also run on each plate. Turbidity (OD_600_) was determined using a SpectraMax iD3 platereader. A minimum of three biological replicates were performed. Any sample well with >70 % growth reduction was considered a hit.

#### Anticancer activity

A549 non-small cell lung cancer cells (ATCC CCL-185) were obtained directly from ATCC and used within 30 passages. A549 cells were maintained in RPMI 1640 medium supplemented with 10 % FBS, 100 U ml^−1^ penicillin and 100 mg ml^−1^ streptomycin. For anticancer testing, cells were seeded at 2000 cells per well in 100 µl of media in 96-well plates and allowed to adhere overnight. Cells were treated with extracts at 50 µg ml^−1^ (1 % DMSO final) or vehicle control for 48 h. Viability was assessed using the sulforhodamine B (SRB) assay protocol [66]. OD was measured using a SpectraMax iD3 platereader (510 nm). Percentage death was calculated by subtracting the background from all wells and setting 0 % growth reduction to vehicle-treated controls. Any well with >70 % growth reduction was considered a hit.

## Results

### Soil characterization

Soil samples from three locations in Utah and four locations in Illinois were collected to examine the biosynthetic potential of *Actinomycetota* from fluorine mines. To evaluate the conditions in each location, we performed an elemental analysis that is summarized in Table 1. The alkaline pH observed for four soils aligns with the characteristics of saline, arid and semi-arid regions where minimal precipitation and high temperatures lead to intense evaporation [67]. Among the examined soils, Topaz Mountain soil emerged as the most inhospitable environment, having chloride, sulphate and nitrate concentrations that exceeded average soil values by 16.8×, 15.1× and 4.5×, respectively [68] (Table 1). Topaz Mountain in Utah’s Juab County is a popular destination for rockhounds, geologists and campers due to its abundant topaz crystals [Al_2_SiO_4_(F,OH)_2_]. The predominant rock type in the Topaz Mountain area is rhyolite, an extrusive igneous rock formed from magma rich in silica, which has led to the discovery of valuable resources such as fluorspar, uranium and beryllium [69].

**Table 1.** Elemental analysis of soils

Soil	Location	Chloride (p.p.m.)	Sulphate **(p.p.m.)**	Nitrate (p.p.m.)	Fluoride (p.p.m.)	pH
Reference*	n/a	145*	350*	19*	585*	Variable
Control	Potting soil	<50	<50	<10	<10	7.5
Topaz Mountain	Utah	2440†	5300†	86†	62	8.31
Bell Hill	Utah	890	123	38	118†	9.65
Yellow Chief	Utah	185	<50	<10‡	< 10‡	8.48
Minerva	Illinois	<50‡	752	< 10‡	44	7.31
Hogg	Illinois	<50‡	<50‡	< 10‡	99	7.03
Spar pile	Illinois	<50‡	<50‡	< 10‡	48	7.05
PMT	Illinois	<50‡	<50‡	22	18	8.33

*The estimated abundance of chemical elements in Earth’s (continental) crust, according to CRC Handbook of Chemistry and Physics [68].

†Highest value in the dataset.

‡Lowest value in the dataset.

Conversely, the soil sample collected from the PMT mine in Illinois had chloride, sulphate and fluoride levels similar to those observed in our control soil, and in some cases, even below the average concentrations (Table 1). The PMT mine is located in the Illinois–Kentucky fluorspar district in eastern Pope County within the Shawnee Forest [70]. It has mainly fluorite (CaF_2_) ore with minor amounts of galena and sphalerite. The mineralization was formed by a mixture of different fluids, including carbonate deposits and NaCl-rich hydrothermal and magmatic fluids connected to ultramafic intrusions. The ultramafic igneous rock provided some fluorine to the ore-forming solutions, fracturing the host rock and stabilizing the pH, producing ore minerals [25,70]. These findings suggest a more favourable environment for bacteria in the PMT mine soil. Interestingly, none of the soil samples examined exceeded the average fluoride content.

### Bacterial biodiversity

**Fig. 1.**
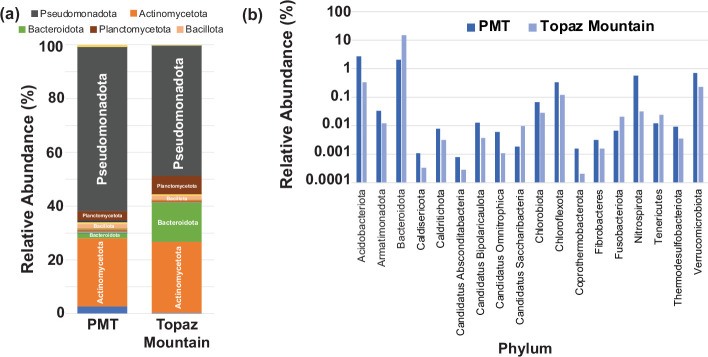
Microbiome composition of soils from PMT and Topaz Mountain mines. (**a**) Relative abundance of bacterial phyla from each soil. Note that only the top five most abundant phyla are indicated. Details of all phyla can be found in Fig. S1 and File S1. (**b**) Relative abundance of phyla that were at least twofold different between PMT (dark blue) and Topaz Mountain (light blue) soils.

Due to the differences observed in the geology and elemental composition of the PMT and Topaz Mountain samples, we conducted a metagenomic analysis to compare the bacterial diversity and explore the potential for NP biosynthesis. This evealed a diverse bacterial population encompassing more than 1700 genera across 40 distinct phyla at each site (Figs 1 and S1, and File S1**,** *Supplementary_Files S4_relative_bacterial_abund* tab). The most prevalent phyla in those environments were *Pseudomonadota*, *Actinomycetota*, *Bacteroidota*, *Planctomycetota* and *Bacillota* (Fig. 1a). The distribution patterns of phyla align with previous studies investigating bacterial communities in soils associated with iron, cadmium and lead–zinc tailing sites [71,74], supporting the consistency of phyla distributions across diverse mining environments.

Another noteworthy aspect of the community composition was the higher relative abundances of *Acidobacteriota*, *Armatimonadota*, *Caldisericota*, *Calditrichota*, Candidatus Absconditabacteria, Candidatus Bipolaricaulota, Candidus Omnitrophica, *Chlorobiota*, *Chloroflexota*, *Coprothermobacterota*, *Nitrospirota*, *Thermodesulfobacteriota* and *Verrucomicrobiota* in the PMT mine, which were around two to nine times higher compared to Topaz Mountain (Fig. 1b). Many of these phyla have been described as members of root endosphere communities [75]. Conversely, the community composition in Topaz Mountain showed higher relative abundances of *Bacteroidota* than in PMT soil. Candidatus Saccharibacteria, *Dictyoglomota*, *Fusobacteriota* and *Planctomycetota* were also more abundant in Topaz Mountain compared to PMT (Fig. 1b). The phyla *Balneolota*, *Chlamydiota*, *Elusimicrobiota*, *Bacillota*, *Ignavibacteriota*, *Rhodothermota* and *Actinomycetota* displayed comparable relative abundances in both the PMT and Topaz Mountain soils (Fig. S1). Notably, the phylum *Actinomycetota* consistently maintained a relative abundance of 25.4 and 26.4 % in the PMT and Topaz Mountain samples, respectively. This underscores the adaptability of *Actinomycetota* to many distinct environments.

## *Actinomycetota* diversity

**Fig. 2.**
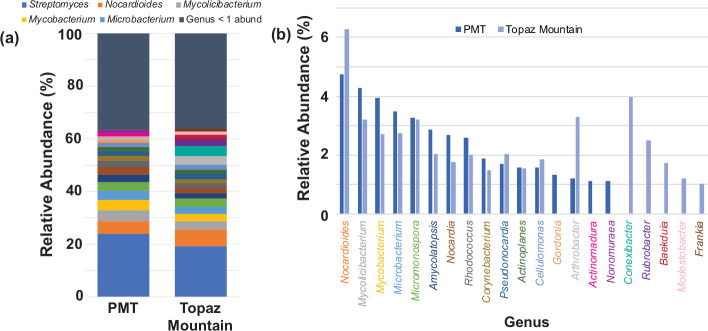
*Actinomycetota* abundance in the Topaz Mountain and PMT soils. (**a**) Relative abundance of *Actinomycetota* genera from each soil. Only the top five genera are indicated. Genus abundance <1 % (abund) includes any unknown taxa. See File S1 for more details. (**b**) Comparison of relative abundance of genera from PMT (dark blue) and Topaz Mountain (light blue) other than *Streptomyces* and those at <1 %. Colours of genus names reflect colours of bars in (a).

To gain deeper insights into the actinomycetota composition within these communities, we conducted an in-depth analysis of the metagenomic data to assess the diversity of this phylum. Our research revealed 222 unique genera, with 22 having relative abundances over 1 % (Fig. 2 and File S1**,** *Supplementary_Files S5_relative_actinomycetota_abundance* tab). Interestingly, while the overall relative abundances of actinomycetota genera were similar between the PMT and Topaz Mountain soils, some genera were exclusive to specific environments. *Gordonia*, *Actinomadura* and *Nonomurea* were only detected in the PMT soil. Conversely, *Conexibacter*, *Rubrobacter*, *Baekduia*, *Modestobacter* and *Frankia* were limited to the Topaz Mountain environment. *Conexibacter, Rubrobacter* and *Baekduia* belong to the subclass *Rubrobacteridae* [76] and are known for their exceptional survival abilities in extreme conditions. These genera are considered exotic micro-organisms and have been recognized as indicator groups for low organic matter presence, frequently isolated from extreme environments [77].

### Metabolic highlights of the actinomycetota community

**Fig. 3.**
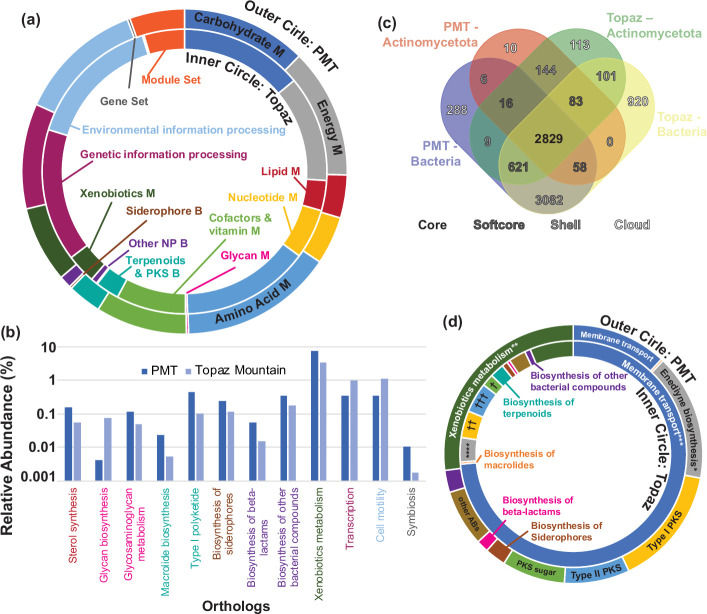
Deciphering metabolic pathways from metagenomic data. (a) *Actinomycetota* metabolic landscape. The graph displays the relative abundance of orthologues from Topaz Mountain (inner circle) and PMT (outer circle), sorted into 13 pathway modules and two signature modules (gene set and module set). M=metabolism; B=biosynthesis; PKS=polyketide synthase. (b) Orthologues with differing abundances. Relative abundance of orthologues from (a) with at least twofold difference in percentage relative abundance. Colours for orthologues indicate the pathway module they belong to, as seen in (a). (c) Comparative analysis of functional orthologues. The totality of orthologues identified for each site were divided into two groups, actinomycetota and bacteria (bacteria not belonging to the phylum *Actinomycetota*), and subsequently categorized into ‘core’, ‘soft-core’, ‘shell’ and ‘cloud’ clusters. (d) Main exclusive actinomycetota pathways. The graph shows the proportion of orthologes for the principal actinomycetota pathways found exclusively in Topaz Mountain and PMT. Certain pathways unique to specific sites are also identified. *Enediyne biosynthesis: kedarcidin, C1027 chromophores; **Xenobiotic metabolism: benzoate degradation; ***Membrane transport/uptake: xylobiose, erythritol, glutamate, galactitol and l-ascorbate; and Membrane trasport/export: lantibiotics; ****Enediyne biosynthesis: polyene macrolides; †Type I PKS: calicheamicin and maduropeptin; ††Type II PKS: ansamycins; PKS sugar biosynthesis: jadomycin, nogalamycin and landomycins.

In addition to exploring the taxa present in the metagenomes, we also explored the metabolic capabilities. To streamline this task, we integrated the taxonomic classification system Kraken2 [78] and the Bayesian Reestimation of Abundance with KrakEN (Bracken) [35] alongside the functional annotation tool EggNOG [38], in combination with the Kyoto Encyclopedia of Genes and Genomes (KEGG) database [79]. We utilized the KEGG mapper tool [80] to perform an extensive *in silico* metabolic reconstruction from our metagenomic data. This integrated approach allowed us to identify functional orthologues and link them to their respective micro-organisms. It also enabled us to extract the information corresponding to the *Actinomycetota* community and construct a comprehensive metabolic map for the PMT and Topaz Mountain environments. Considering both soil samples, we discovered approximately 8280 functional orthologues (Fig. 3a). Interestingly, 48 % of these orthologues were present in the actinomycetota population, which accounts for a quarter of the bacterial communities (Fig. 1). Using the KEGG-DB classification as a framework, we organized our functional orthologues into 48 pathways, which were further categorized into 13 pathway modules (functional units of gene sets in metabolic pathways, including molecular complexes) and two signature modules (functional units of gene sets that characterize phenotypic features) (Fig. 3a, b, Table S3 and File S1, *Supplementary_Files S6* tabs).

As expected, both environments displayed similar levels of central metabolism and other carbohydrate degradation pathways, including galacturonate, galactose and glycogen. Interestingly, they exhibited higher abundance values for orthologues involved in trehalose biosynthesis (File S1, *Supplementary_Files S6_Other_carb_metabolism* tab). Trehalose, a universal stress molecule, protects cells and biomolecules from various environmental stresses, including high osmolarity, heat, oxidation, desiccation and freezing, as well as serving as a carbon source in challenging environments. Critical metabolic pathways, including methane and nitrogen metabolism, were found to have similar abundances and representation in both the PMT and Topaz Mountain environments (Fig. 3a). Sulphur metabolism also showed comparable levels, except for the assimilatory sulphate reduction pathway and cysteine metabolism. The Topaz Mountain environment exhibited increased abundance in these pathways (File S1, *Supplementary_Files S6_Sulfur_metabolism* tab and *Supplementary_Files S6_Cysteine and methionine metabol* tab), suggesting adaptive coping strategies for its elevated sulphate levels. Places like Topaz Mountain are subject to harsh conditions, including desiccation, high UV irradiation and exposure to toxic compounds, which induce DNA damage [81]. Notably, the Topaz Mountain actinomycetota community has an increased abundance of genetic information processing orthologues, especially in protein synthesis and DNA replication/repair, to combat these conditions (Fig. 3a, b).

The environmental information processing modules show similar abundances between PMT and Topaz Mountain, except for membrane transport (Fig. 3). Topaz Mountain has higher abundances in PTS-type substrate uptake systems, suggesting nutrient scarcity (File S1, *Supplementary_Files S6_Membrane transport* tab). Additionally, actinomycetota in Topaz Mountain possess quinolone and lantibiotic exporters, indicating potential NP biosynthesis (File S1). Regarding secondary metabolism, our analysis revolved around three modules: terpenoid and polyketide biosynthesis, non-ribosomal peptide synthesis (NRPS) and other secondary metabolites. Notably, functional orthologue abundance across sub-modules showed no significant environmental differences, except for terpenoid backbone biosynthesis-related pathways where Topaz Mountain demonstrated significantly higher abundance (Fig. 3). Finally, our analysis revealed a slightly higher orthologue abundance of drug resistance and metabolic capacity subsets in the PMT environment (Fig. 3).

### Insights into *Actinomycetota* metabolic adaptations

Metagenomics provides community profiles and can define sub-populations, offering novel insights into diverse community interactions and revealing important roles of their inhabitants inside the community. To better understand metabolic behaviour, we examined the genetic diversity and evolutionary processes of actinomycetota populations within a taxon found in these environments by comparing functional orthologues. During the metabolic reconstruction, we compared actinomycetota orthologues with the broader bacterial community in each environment. In the field of genomic research, certain terms such as ‘core’, ‘softcore’, ‘shell’ and ‘cloud’ are used to describe the distribution of genes within a pre-defined set of genomes. In our research, we use this classification to identify the distribution of genes, expressed here as functional orthologues, within the phylum *Actimomycetota* compared with the rest of the bacteria present in our metagenomes. These terms, used during metagenomics analysis, are often referred to as pan-metagenomics analysis [82,84]. The objective of this comparison is to establish a genetic hierarchy that will enable us to identify functional orthologues in the context of secondary metabolism. We grouped genes into four categories based on their presence in the datasets: ‘core’ (those orthologues present in every dataset), ‘softcore’ (those orthologues present in at least three of the datasets), ‘shell’ (those orthologues present two of the datasets), and ‘cloud’ (those orthologues present in only one of the datasets, Fig. 3c). Specifically, we analysed 8280 orthologues spanning both metagenomes and found the following distribution: 34.4 % core, 9.4 % softcore, 40.4 % shell and 16 % cloud genes (Fig. 3c). Within the PMT and Topaz Mountain environments, *Actinomycetota* displayed 237 and 282 unique orthologues, respectively, spanning across the softcore, shell and cloud classifications. Only a few shared shell orthologues were detected between actinomycetota and bacteria from both PMT and Topaz environments, underscoring the distinct specialization of actinomycetota populations. Of particular interest, we documented a noteworthy cluster of genes categorized as ‘shell’ and exclusive to actinomycetota, prominently featuring fatty acid synthase genes (File S1). These genes may possess a specialized role in polyketide synthesis. Type I and type II polyketide synthases (PKSs) share significant similarity with fatty acid synthases (FASs) [85,87]. This similarity complicates the determination of whether the annotation of these orthologues pertains to PKS domains mistakenly labelled as FAS or truly FAS enzymes, acting either in traditional fatty acid synthesis or in biosynthesis of natural products. However, the presence of these orthologues only in the actinomycetota, which are characterized by a notable presence of NP BGCs, suggests these genes are probably participating in secondary metabolism. This undoubtedly sparked interest in molecular evolution that other papers have previously addressed very well [88,89]. A comprehensive analysis of this aspect will be addressed in a future publication.

We identified key modules in shell and cloud orthologues unique to actinomycetota, which were involved in membrane transport and NP biosynthesis in both environments. Topaz actinomycetota showed sevenfold higher membrane transporter orthologues than PMT for this entry (Fig. 3d), indicating specialization in substrate uptake. Abundant actinomycetota orthologues observed in xylobiose and erythritol metabolism suggest an active pentose phosphate pathway (PPP) in Topaz Mountain, indicating a preference for these carbohydrates by micro-organisms in this environment. The PPP in Topaz Mountain probably maintains carbon homeostasis, precursor supply for nucleotide/amino acid biosynthesis, and combats stress [90]. The metagenomic analysis also underscores the prevalence of purine and pyrimidine metabolism in Topaz actinomycetota (File S1, *Supplementary_Files S6_Purine Metabolism* tab and *Supplementary_Files S6_Pyrimidine Metabolism* tab), which aligns with an active PPP being responsible for furnishing essential carbon and nitrogen precursors [91]. Comparative analysis unveiled a higher abundance of exclusive actinomycetota pathways associated with the biosynthesis of antitumour and antibiotic compounds in the PMT environment (Fig. 3d and File S1). A notable discovery in the Topaz Mountain environment was the presence of distinct groups of cloud orthologues associated with various secondary metabolism pathways. These pathways encompass polyene macrolides, calicheamicin, ansamycins, jadomycins, nogalamycins and landomycins, highlighting the environment’s potential for diverse and unique secondary metabolite production (Fig. 3d and File S1).

### Isolation of individual strains and bioactivity screening

To discover actinomycetota capable of producing novel bioactive NPs, we utilized selective media to isolate these micro-organisms from the PMT and Topaz Mountain environments. This methodology resulted in a diverse collection of 21 strains, subsequently identified via 16S rRNA gene sequencing (Table S1). As anticipated, most isolated bacteria were affiliated with the phylum *Actinomycetota*, originating primarily from the genus *Streptomyces* along with a few ‘rare’ actinomycetota. To probe their biosynthetic abilities, we grew each strain on five culture media (R4, SFM, ISP2, ISP4 and GYM). After pooling the supernatant from the media, we screened for antibiotic and anticancer activity. Five strains (PMTA, PMTD, PMTG, S1A and BPP2) showed antibiotic activity against at least one ESKAPE pathogen. The PMTA supernatant also demonstrated anticancer activity against lung cancer cells. For the strains that showed bioactivity, we performed whole-genome sequencing. We also sequenced strain BPT11 as it was one of the few strains isolated from the Topaz Mountain environment (Table S1).

### Evolutionary genome mining

We obtained high-quality whole-genome sequencing data for six strains using Illumina and Nanopore-integrated sequencing technologies (Tables 2 and S4). These data were subjected to a comprehensive genome mining workflow, encompassing genome assembly, taxonomic assignment, and the identification and networking of BGCs. Our phylogenomic analysis, utilizing the 19 155 type strains from the TYGS database, revealed that four of our strains represent novel *Streptomyces* species (Fig. S2) with three originating from PMT and one from Topaz Mountain. Based on the analysis of average nucleotide identity (ANI), it was found that *Streptomyces wuyuanensis*, *Streptomyces* sp. ZYC3, *Streptomyces spectabilis* and *Streptomyces anulatus* are the closest strains to the novel PMTA, PMTD, PMTG and S1A strains, with identities of 92.4, 94.53, 89.03 and 94.23 %, respectively. The identities of the remaining two (BPP2 and BPT11), one from each site, matched with the previously described strains: 99.22 % with *Streptomyces libani* [92] and 97.8 % with *Micrococcus yunnanensis* [93] (Fig. S2).

**Table 2.** Information regarding the isolated strains

Strain	Location	No. of contigs	Genome size (Mb)	Total BGCs	BiNI score
*Streptomyces libani* BPP2	PMT	64	9.0	32	886
*Streptomyces* sp. PMTA	PMT	34	8.1	24	951
*Streptomyces umbrinus* PMTD	PMT	155	11.2	39	1002
*Streptomyces* sp. PMTG	PMT	37	9.7	42	1305
*Streptomyces* sp*.* S1A	Topaz	6	8.3	32	1086
*Micrococcus yunnanensis* BPT11	Topaz	82	2.7	6	770

**Fig. 4.**
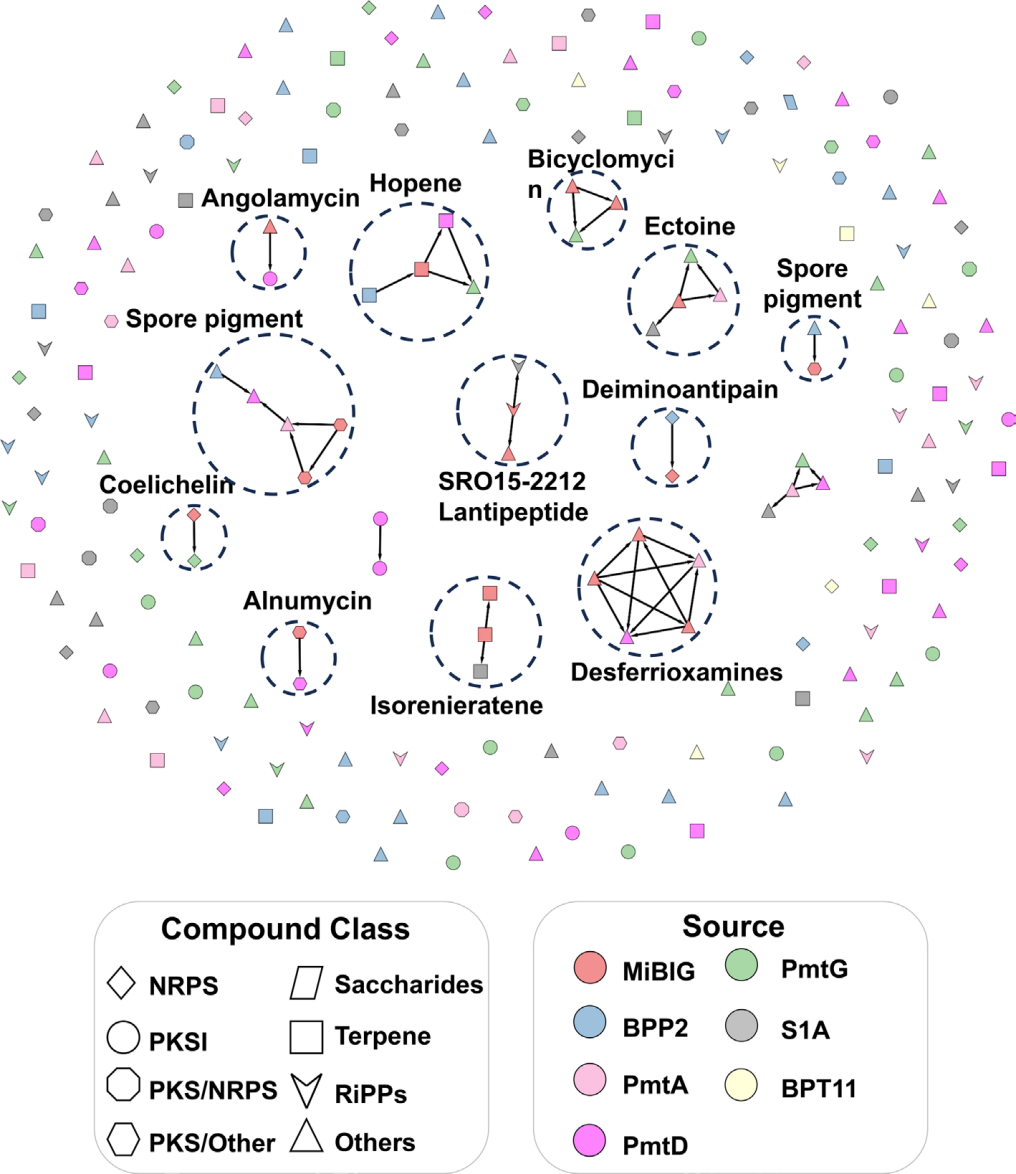
Big-SCAPE network with 0.3 cut-off BGC similarity value. BGC clusters and singletons from six indicated strains. Clusters with BGCs similar to known BGCs in the MiBIG database are indicated (red is BGC from MiBIG). Shapes indicate the type of BGC. Colour indicates the source of the BGC.

Using antiSMASH, we identified 175 BGCs from the six strains. Among these, *Micrococcus yunnanensis* had the lowest count, with only six BGCs, while the PMTG strain had the highest count with 42 BGCs (Table 2 and File S1, *Supplementary_Files S7_Antismash_predicted_BGCs* tabs). We utilized BiG-SCAPE software [54] to compare these BGCs to previously described BCGs listed in the MiBIG database [55]. Interestingly, our BGC network revealed only 18 significant relationships, comprising 12 different NPs, with 152 singletons representing novel BGCs (Fig. 4). To further ascertain the novelty of the biosynthetic potential encoded by each strain, we determined their BiNI scores [57]. BiNI scores are calculated by comparing BGCs found in actinobacteria in public databases, with higher scores indicating greater diversity. As anticipated, we found that the strains most closely related to other bacterial strains (i.e*. S. libani* and *M. yunnanensis* strains) had the lowest BiNI score, 886.28 and 769.66, respectively. Conversely, PMTG and S1A strains had the highest scores, 1304.71 and 1086.43, respectively, suggesting that they have the most unique BGCs (Table 2).

### Metabolomics of novel strains

**Fig. 5.**
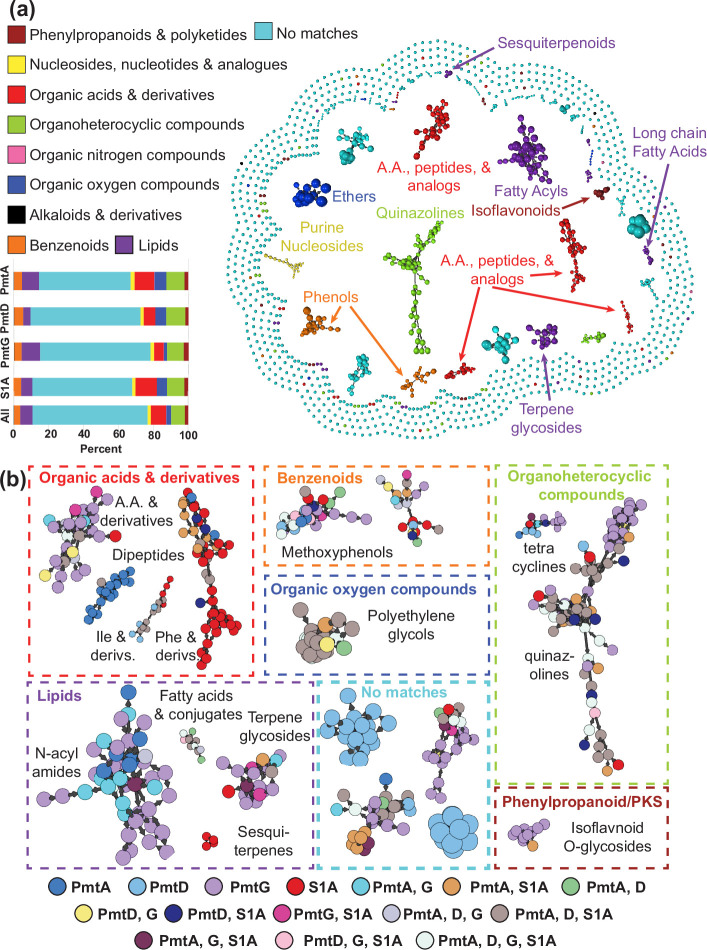
MolNET-enhanced GNPS network. (a) A chemical classified network obtained with the MolNetEnhancer tool. Each colour represents a unique chemical superfamily, while internal labels within the network specify the chemical subclass. Size of node indicates relative ion abundance in the network. Following the same colour code, the bars at lower left denote the relative abundance of each superfamily in each strain and as a whole. (b) Most significant sub-networks from (a) presented individually. They are colour-coded with a 15-colour scheme to represent their bacterial origin. Edges in the graph show the direction from the parent to the fragment ion.

We analysed the metabolomic profile of the four newly identified strains with the highest BiNI values. As before, we obtained supernatants from the bacteria grown on the same five media and conducted untargeted LC-MS analysis. The data were then used to build metabolic networks via the GNPS platform [94]. By combining MolNetEnhancer from GNPS [48] and the Structural similarity Network Annotation Platform or Mass Spectrometry (SNAP-MS) tool from the NP-Atlas platform [51], we constructed a robust 1534-node network. This network was engineered to explore chemical attributes, effectively serving as the foundation for our NP identification endeavours. Using the ‘superclass’ category to structure the metabolic network, we discerned ten distinct metabolite groups, including those with no matches to molecules in the GNPS library (Figs 5 and S3–S8).

Surprisingly, 66 % of the metabolites in our network did not fit into a chemical group within the GNPS classification (Figs 5a and S7–S8). While we would expect based on our BiNI scores to have many new molecules, we would not necessarily expect to have molecules that are unable to fit into these broad chemical classifications. Of the remaining 34 % of the metabolites, the most commonly produced were organic acids, organoheterocyclic compounds and lipid molecules (8.8, 7.8 and 7 % of the overall metabolites, respectively). The remaining 10.4 % are distributed among the other six chemical groups. When we examined the various strains individually, similar distributions were observed (Fig. 5a).

Within the organic acids and derivatives category, we identified four main subnetworks: alpha amino acids (A.A.), isoleucine and its derivatives, phenylalanine and its derivatives, and the dipeptide subnetwork. Notably, the PmtG strain predominantly produces alpha-amino metabolites but no isoleucine and derivatives molecules. In contrast, the dipeptide subnetwork exclusively features metabolites produced by the PmtA strain. Furthermore, the phenylalanine and derivatives subnetwork primarily includes metabolites produced by the S1A strain, with more than half of the metabolites being unique to this particular strain (Fig. 5b). Utilizing the SNAP-MS tool [51], we predicted the identity of some secondary metabolites, such as asukamycins and nocapyrones, within the phenylalanine and derivatives subnetwork produced by the PmtA and S1A strains (Fig. S3).

Within the benzenoid superclass, we identified two prominent subclusters predominantly composed of methoxyphenols produced by all strains (Figs 5b and S5). In contrast, the ether superclass revealed a singular subcluster primarily composed of polyethylene glycols produced mostly by the PmtA, PmtD and S1A strains. The lipid and lipid-like molecules superclass exhibited four significant subclusters: *N*-acyl-amides, fatty acids and conjugates, terpene glycosides, and sesquiterpenoids (Figs 5b and S6). Notably, the sesquiterpenoids subcluster exclusively consisted of metabolites derived from the S1A strain, while the *N*-acyl-amides subcluster was predominantly associated with the PmtD and PmtG strains.

The organoheterocyclic compounds superclass displayed two distinct subclusters encompassing molecules from all strains. The first subcluster, comprising quinazolines, exhibited the largest number of nodes of the network, while the second subcluster consisted of tetracyclines. In addition, within the phenylpropanoid and polyketides superclass, we identified a subcluster composed primarily of isoflavonoid *O*-glycosides. Interestingly, this subcluster was predominantly associated with the PmtG strain, suggesting its unique biosynthetic capability in producing these compounds.

Remarkably, only 12.9 % of the network’s metabolites exhibited potential spectral matches with molecules in the GNPS libraries, highlighting the exceptional novelty and untapped potential offered by these strains. Upon further investigation of these molecules, our analysis unveiled four prominent subclusters with the highest number and concentration of unmatched compounds. Intriguingly, two of these subclusters were exclusively associated with the *Streptomyces* sp. PmtD strain (Fig. 5b). By utilizing the capabilities of the SNAP-MS tool, we predicted that some of the metabolites may be analogues of known NPs such as serrawettin, kahalalide and rapamycin (Fig. S7). This discovery strongly hints at the existence of macrolides and/or lipopeptides produced via hybrid PKS-NRPS systems. Notably, antiSMASH analysis yielded a hit revealing the presence of a PKS-NRPS with 75 % similarity to the BGC for the cyclic hexadepsipeptide polyoxypeptin [95], further supporting this finding. Using SNAP-MS we also identified additional potential molecules within a distinct secondary subcluster, exclusively consisting of PmtD elements absent from the GNPS library. Noteworthy candidates include the siderophores nocobactin and nocardimicin, along with analogues of the macrolides cirramycin, M-4365 and rosamicin (Fig. S7).

In Figs S3–S8, we present approximately 50 potential matches from our metabolomic findings with the NP-Atlas database, encompassing only about 3 % of our entire metabolic network. Considering that a substantial 66 % of the network does not match with any known chemical group within the GNPS libraries, it is reasonable to speculate that novel NPs await discovery. This alignment is consistent with our genome mining data, where only 19 BGCs (10 % of the total) exhibit high similarity values with the BGCs in the MIBiG database (Fig. 4 and File S1, *Supplementary_Files S7_Antismash_predicted_BGCs* tabs). Notably, the majority of potential BGCs we identified exhibit similarity levels below 80 % according to antiSMASH results. Our preliminary antibiotic and anticancer screenings, coupled with the genomic and metabolomic characterization, provide compelling support for novel molecule discovery from bacteria from fluoride mines.

## Discussion

While scientists have studied bacteria from soil ecosystems for many years, many questions surrounding the types of bacteria likely to be found in certain environments still exist. In a significant study conducted in 2018, researchers carefully analysed soil samples taken from 237 different locations across six continents, covering 18 countries and nine distinct ecosystems [96]. Of the many different types of bacteria, a small fraction – approximately 2 % – comprising 511 phylotypes across ten phyla emerged as remarkably prevalent, collectively constituting nearly half of the global soil bacterial community. This revelation underscores the paradoxical simplicity underlying the immense complexity of soil microbial ecosystems, where a select few taxa yield disproportionate influence. However, it also highlights the vast diversity observed across the planet and how much we have yet to learn about bacterial adaptation to differing environments. After analysing these findings and comparing them with our own dataset, we have come across some interesting insights. While the two dominant phyla in our datasets (*Pseudomonodata* and *Actinomycetota*) are consistent with those previously observed in soils across the globe, 30 of the 40 observed phyla are deemed relatively uncommon, their presence scarcely felt on a global scale (see Fig. S9). Delving deeper into the *Actinomycetota* group, the global analysis unveils a diverse assemblage of 148 phylotypes. Of these phylotypes, 47 have genera indicated, with 26 distinct genera being identified. Notably, only 11 of the 22 major genera identified in our samples align with the roster of ubiquitous soil bacteria (see Fig. S9). Notably, specific phylotypes belonging to the genera *Amycolatopsis*, *Actinoplanes* and *Streptomyces* demonstrated prevalence in dry forest ecosystems like PMT. Conversely, a phylotype from the genus *Frankia* emerged as dominant in low-pH environments. Meanwhile, the remaining widespread phylotypes identified in our study further demonstrate bacterial diversity across differing environmental conditions. Among the remaining 11 genera classified as uncommon, two are exclusive to PMT (*Actinomadura* and *Nonomurea*), while two others are unique to Topaz Mountain (*Baekduia* and *Conexibacter*). This comparison reveals the complex interaction between soil composition and bacterial communities, highlighting distinct microbial populations in localized niches within a global framework.

Many of the predominant phyla in the PMT mine have been described as members of root endosphere communities [75]. Considering that the PMT mine is located within the Shawnee National Forest, a deciduous hardwood forest predominantly characterized by oak–hickory stands [97], it is plausible to infer a presence of vegetative matter and, hence, potential associations between the bacterial community and plant roots in the PMT mine soil. Conversely, the community composition in Topaz Mountain showed higher relative abundances of *Bacteroidota* than in PMT soil and those found in the global study [96]. *Bacteroidota* are known for their versatile metabolic capabilities and have been associated with various ecological niches, including soils, sediments and aquatic systems [98,100]. This group of bacteria has also been described as inhabitants of extreme environments such as the Spotted Lake in British Columbia, Canada, a hypersaline lake with excessive sulphate salts [101]. *Bacteroidota* have garnered attention for their well-documented metabolic capacities and are already acknowledged as a significant reservoir of secondary metabolites [102].

Interestingly, the PMT environment exhibited a slightly higher relative abundance of carbon fixation pathways than Topaz Mountain. Carbon fixation is a vital process wherein organisms convert atmospheric carbon dioxide into organic compounds, playing a crucial role in the carbon cycle and ecosystem productivity [103]. Traditionally, only a few autotrophic lineages were believed to be involved in this process, with *Actinomycetota* often overlooked. However, recent discoveries have shed light on actinomycetota metagenome-assembled genomes (MAGs) from hot springs and hypersaline lakes, revealing the presence of gene sets associated with the Wood–Ljungdahl pathway (WLP) [104,105], a critical pathway for carbon fixation. Our metabolic reconstruction strongly supports the presence of the WLP in both actinobacterial communities (File S1, *Supplementary_Files S6_Carbon fixation* tab), indicating this pathway could be more widespread in *Actinomycetota* than previously acknowledged. However, it is crucial to note that direct evidence demonstrating the utilization of the WLP for carbon fixation by *Actinomycetota* is currently lacking. This knowledge gap highlights the significance of this study, as it provides valuable actinobacterial orthologue sequences for evolutionary analyses related to this pathway.

Ore-forming environments remain largely unexplored for their ability to produce NPs. Our research indicates these environments offer a rich source of microbial diversity and novel NPs. The metagenomic analysis of the PMT and Topaz Mountain environments has unveiled pronounced differences, including variations in the presence and abundance of bacterial taxa and divergent metabolic pathways, particularly within the realm of secondary metabolism. A high degree of metabolic adaptation was specifically evident in the *Actinomycetota* community, where distinct pathways, mostly from secondary metabolism, prevail. Genomic and metabolomic analyses of individual strains isolated from these environments further support their biosynthetic potential, with a plethora of unknown BGCs and metabolites that are absent from the most comprehensive databases.

There is still much to learn about *Actinomycetota* pangenomics for the discovery of adaptations reflected in BGC composition. One promising method for exploring these topics is metagenomics. However, this task presents a challenge due to the remarkable ability of *Actinomycetota* to exchange DNA, which has greatly influenced its survival in diverse environments since ancient times. Recent comparative studies show that the genetic makeup of *Actinomycetota* species and populations undergoes continuous change due to widespread horizontal gene transfer (HGT), driven by factors such as local conditions and interactions with other microbes [106]. *Actinomycetota* utilize various mechanisms to acquire DNA, including mobile genetic elements, dsDNA conjugation and phages. This acquired genetic material can be integrated into BGCs, expanding the chemical capabilities of a species [107]. Understanding these mechanisms will enable the community to learn more about the ecological adaptations of micro-organisms and how to harness their potential.

Integrating genomic and metabolomic data (metabologenomics) holds vast potential for discovering NPs, yet it faces significant challenges due to the complexity of biological systems and current methodological limitations. Factors such as incomplete functional annotation of proteins and molecules hinder clear associations between genomic information and metabolite production, particularly in complex microbial communities. Moreover, discrepancies in experimental protocols, data processing methods and formats impede data comparison and integration, requiring standardized workflows and quality control measures. Dealing with the immense volume of data presents another hurdle, as the inclusion of low-quality data may compromise analysis accuracy, necessitating careful filtering without excluding relevant information. Standardizing sample information and metadata remains a challenge despite efforts through domain-specific ontologies and initiatives. Promising advancements in computational tools like the Paired Omics Data Plantform, mmvec and MelonnPan offer solutions [108,110], yet manual exploration and experimental validation are indispensable for understanding candidate relevance and functionality. Currently, we are taking a comprehensive approach to NP discovery by combining computational analysis with manual exploration and experimental validation. It would be too speculative to claim specific links between BGCs and metabolites observed in this study without further experimental data. However, these data do illuminate the vast realm of metabolites that remain hidden within harsh and inhospitable environments. This in turn underscores the critical importance of further analysis from a metabologenomic perspective and the need to further explore these unique environments for NP discovery.

## supplementary material

10.1099/mgen.0.001253 Uncited Supplementary Material 1.

10.1099/mgen.0.001253 Uncited Supplementary Data Sheet 1.

## Abbreviations

ACN: acetonitrile
ANI: average nucleotide identity
BCG: biosynthetic gene cluster
dDDH: digital DNA–DNA hybridization
eDNA: environmental DNA
FAS: fatty acid synthase
GBDP: Genome blast Distance Phylogeny
GCF: Gene Cluster Family
gDNA: genomic DNA
GNPS: Global Natural Product Social Molecular Networking
MAG: metagenome-assembled genome
NCBI: National Center for Biotechnology Information
NP: natural product
NRPS: non-ribosomal peptide synthesis
PKS: polyketide synthase
PPP: pentose phosphate pathway
SNAP: Structural Similarity Network Annotation Platform
WLP: Wood–Ljungdahl pathway
