# Supplementary material for: Actinomycetota bioprospecting from ore-forming environments
Source: Microb Genom. 2024 May 14;10(5):001253. doi: 10.1099/mgen.0.001253 (PMC11165632; doi:10.1099/mgen.0.001253)
Supplement: Uncited Supplementary Material 1. [file mgen-10-01253-s001.pdf]

Supplementary Information

Actinomycetota bioprospecting from ore-forming environments

César Aguilar[1], Amir Alwali[1], Madeline Mair[1], Lorena Rodriguez-Orduña[2], Haydeé Contreras-Peruyero[3], Ramya Modi[1], Nelly Sélem-Mojica[3], Cuauhtemoc Licona-Cassani[2], Elizabeth Parkinson[1,4*]

[1]Department of Chemistry, Purdue University, West Lafayette, IN, 47907 USA. [2]Tecnológico de Monterrey, Escuela de Ingeniería y Ciencias, Monterrey, México. [3]Centro de Ciencias Matemáticas, UNAM, Morelia, Michoacán, México. [4]Department of Medicinal Chemistry and Molecular Pharmacology, Purdue University, West Lafayette, IN, 47907 USA.

*Corresponding author: eparkins@purdue.edu

Supplementary Information

Table of Contents

Table S1: Strains Isolated and Bioactivity Observed 3

Table S2: Databases employed during Classical Molecular Networking 4

Table S3: KEGG-DB classification of orthologs 5

Table S4: Genome statistics for isolated strains 5

Figure S1. Relative Abundance of Phylum in PMT versus Topaz Mountains. Relative abundance of phyla in PMT (dark blue) and Topaz Mountain (light blue). Note that the axis is on a logarithmic scale. 6

Figure S2. Phylogenomic tree. Taxonomic identification of the six strains sequenced in this study (identified in bold). Within the tree, the species, subspecies, relative GC content, and delta statics of each strain, represented by colored squares, were included. For the species and subspecies, these colors are randomly assigned and simply indicate which branch each one belongs to. For GC content, the darker the blue indicates the higher percentage GC. For the delta statistics, this value demonstrates how closely species are related with lighter colors indicating less related (70% or lower for the digital DNA-DNA hybridization (dDDH) between the genomes from the strains sequenced here and the selected typestrain genomes in most cases). Each strain's relative genome size and relative protein count are shown by black and orange bars, respectively. Bootstrap values for each branch are shown in blue. 7

Figure S3. Secondary metabolites predictions for organic acids and derivatives. MolNET-enhanced GNPS networks with potential molecules identified by the SNAP-MS software. Colors indicate the strain of origin. 8

Figure S4. Secondary metabolites predictions for dipeptides. MolNET-enhanced GNPS networks with potential molecules identified by the SNAP-MS software. Colors indicate the strain of origin. 9

Figure S5. Secondary metabolites predictions for benzenoids. MolNET-enhanced GNPS networks with potential molecules identified by the SNAP-MS software. Colors indicate the strain of origin. 10

Figure S6. Secondary metabolites predictions for lipid and lipid-like molecules. MolNET-enhanced GNPS networks with potential molecules identified by the SNAP-MS software. Colors indicate the strain of origin. 11

Figure S7. Secondary metabolites predictions for no matches to chemical superfamily, Part 1. MolNET-enhanced GNPS networks with potential molecules identified by the SNAP-MS software. Colors indicate the strain of origin. 12

Figure S8. Secondary metabolites predictions for no matches to chemical superfamily, Part 2. MolNET-enhanced GNPS networks with potential molecules identified by the SNAP-MS software. Colors indicate the strain of origin. 13

Figure S9. Comparison of observed phyla and genera from global locations to those observed in this study. 14

Supplemental References 15

Supplementary Information

Table S1: Strains Isolated and Bioactivity Observed

Name	Place	Identity (16S Seq)	Media**	Bioactivity***
2A	PMT, IL	*Cupriavidus sp.*	SCA	None
2B		*Klebsiella sp.*	SCA	None
3_5_1		*Streptomyces sp.*	SIM	None
3_5_16		*Streptomyces sp.*	SIM	None
BPP2*		*Streptomyces libani*	SIM	Antibacterial
3_5_3		*Streptomyces sp.*	SIM	None
3_5_4		*Streptomyces sp.*	SIM	None
Pmt-A*		*Streptomyces sp.*	SNA	A549 (cancer cell line), *B.subtilis* 6633 (wt)
Pmt-B		*Streptomyces sp.*	SNA	None
Pmt-C		*Streptomyces sp.*	SNA	None
Pmt-D*		*Streptomyces sp.*	SNA	*S.aureus* 29213 (wt)
Pmt-E		*Streptomyces sp.*	SNA	None
Pmt-F		*Streptomyces sp.*	SNA	None
Pmt-G*		*Streptomyces sp.*	SNA	*S.aureus* 29213 (wt), *S.aureus* NRS3 (R)
Pmt-H		*Streptomyces sp.*	SNA	None
Pmt-I		*Streptomyces sp.*	SNA	None
Pmt-J		*Streptomyces sp.*	SNA	None
Pmt-K		*Variovorax sp.*	HA	None
Pmt-L		*Kribbella sp.*	HA	None
S1A*	Topaz Mtn, Utah	*Streptomyces sp.*	SNA	*P.aeruginosa* PAO1 (wt), *S. aureus* 29213 (wt), A549 (cancer cell line)
BPT11*		*Micrococcus yunnanensis*	SIM	None

*Strains sequenced
**Media used for strain isolation
***Bioactivity indicates at least 70% reduction in cell growth when treated with 50 µg/mL extract and compared to DMSO control.
Wt = wild type; R = antibiotic resistant

Supplementary Information

Table S2: Databases employed during Classical Molecular Networking

	**Database**
**1**	Berkeley-LAB
**2**	Dereplicator_identified_library
**3**	Birmingham-UHPLC-MS-NPOS
**4**	GNPS-COLLECTIONS-PESTICIDES-POSITIVE
**5**	GNPS-NIH-NATURALPRODUCTSLIBRARY_ROUND2_POSITIVE
**6**	GNPS-NIH-NATURALPRODUCTSLIBRARY
**7**	GNPS-NIH-SMALLMOLECULEPHARMACOLOGICALLYACTIVE
**8**	LDB_POSITIVE
**9**	MassBank
**10**	MassBankEU
**11**	MMV_POSITIVE
**12**	MONA
**13**	RESPECT

Supplementary Information

Table S3: KEGG-DB classification of orthologs

			Relative abundance (Normalized Value)	
			Pmt2	Topaz Mountain
Pathway Modules	*Carbohydrate metabolism*	Central carbohydrate metabolism	0.043902439	0.048625726
		Other carbohydrate metabolism	0.050008185	0.057666967
	Energy metabolism	Carbon fixation	0.037616631	0.030577633
		Methane metabolism	0.015976428	0.015429118
		Nitrogen metabolism	0.013782943	0.012749621
		Sulfur metabolism	0.007693567	0.00668774
		ATP synthesis	0.022245867	0.026286035
	Lipid metabolism	Fatty acid metabolism	0.024750368	0.019519891
		Sterol synthesis	0.00116222	0.000430535
		Lipid metabolism	0.006351285	0.006149914
	Nucleotide metabolism	Purine metabolism	0.021591095	0.026060451
		Pyrimidine metabolism	0.014372238	0.016955937
	Amino acid metabolism	Serine and threonine metabolism	0.011147487	0.014738611
		Cysteine and methionine metabolism	0.013570142	0.009499286
		Branched-chain amino acid metabolism	0.018906531	0.01738097
		Lysine metabolism	0.019332133	0.018722094
		Arginine and proline metabolism	0.007677198	0.008226937
		Polyamine biosynthesis	0.005794729	0.003286098
		Histidine metabolism	0.004992634	0.005242076
		Aromatic amino acid metabolism	0.010574562	0.011433256
		Other amino acid metabolism	0.022884269	0.022419471
	Glycan metabolism	Glycan biosynthesis	3.27386E-05	0.000583217
		Glycosaminoglycan metabolism	0.000883942	0.000364511
		Lipopolysaccharide metabolism	0.000720249	0.000757907
	Cofactors & vitamin metabolism	Cofactor and vitamin metabolism	0.068521853	0.060213039
	Biosynthesis of terpenoids and polyketides	Terpenoid backbone biosynthesis	0.010738255	0.011927065
		Macrolide biosynthesis	0.000180062	3.98898E-05
		Enediyne biosynthesis	0.004206908	0.003085273
		Type I polyketide structures	0.003322966	0.000779915
		Type II polyketide biosynthesis	0.001997054	0.001193944
		Polyketide sugar unit biosynthesis	0.004796202	0.003601091
	Biosynthesis of NRPs	Biosynthesis of siderophores	0.001767883	0.000881703
	Biosynthesis of other secondary metabolites	Biosynthesis of beta-lactams	0.000409232	0.000115543
		Biosynthesis of other antibiotics	0.006253069	0.003823923
		Biosynthesis of other bacterial compounds	0.002619087	0.001308112
	Xenobiotics metabolism	Xenobiotics metabolism	0.057227042	0.025482736
	Genetic information processing	Transcription	0.002586348	0.007403006
		Translation	0.031068915	0.037799071
		Folding, sorting and degradation	0.016844001	0.028188368
		Replication and repair	0.028449828	0.041416667
	Environmental information processing	Membrane transport	0.037944017	0.05599847
		Signal transduction	0.021427402	0.020906407
		Cellular community	0.032362089	0.032504725
		Cell motility	0.002684564	0.008262701
Signature Modules	*Gene set*	Pathogenicity	0.000409232	0.000338376
		Drug resistance	0.001505975	0.001079777
		Symbiosis	8.18465E-05	1.37551E-05
	Module set	Metabolic capacity	0.041201506	0.035032916

Table S4: Genome statistics for isolated strains

Seq type	ID	bases	genome length	coverage	# Contigs	# reads Illumina	# reads nanopore	N50	L50	% GC	completeness %	contamination %
hybrid	*Streptomyces nov. sp*. PmtA	1.8E+09	8.1E+06	225	34	5.0E+06	3.9E+05	6.4E+05	5	72.3	93.4	10.4
hybrid	*Streptomyces nov. sp*. PmtD	2.0E+09	1.1E+07	178	55	5.8E+06	6.0E+05	2.2E+05	16	70.2	93.4	10.4
hybrid	*Streptomyces nov*. sp. PmtG	2.2E+09	9.7E+06	226	37	4.3E+06	1.0E+06	8.5E+05	4	72.5	96.2	6.6
hybrid	*Streptomyces nov. sp.* S1A	3.3E+09	8.3E+06	393	6	4.6E+06	2.0E+06	1.6E+06	2	71.5	83	7.5
hybrid	*Streptomyces libani* BPP2	1.5E+09	9.0E+06	169	64	2.9E+06	9.4E+05	2.8E+05	10	71.1	91.5	8.5
Illumina	*Micrococcus yunnanensis* BPT11	2.6E+09	2.7E+06	959	82	1.0E+07	0.0E+00	1.2E+05	7	72.8	97.2	2.8

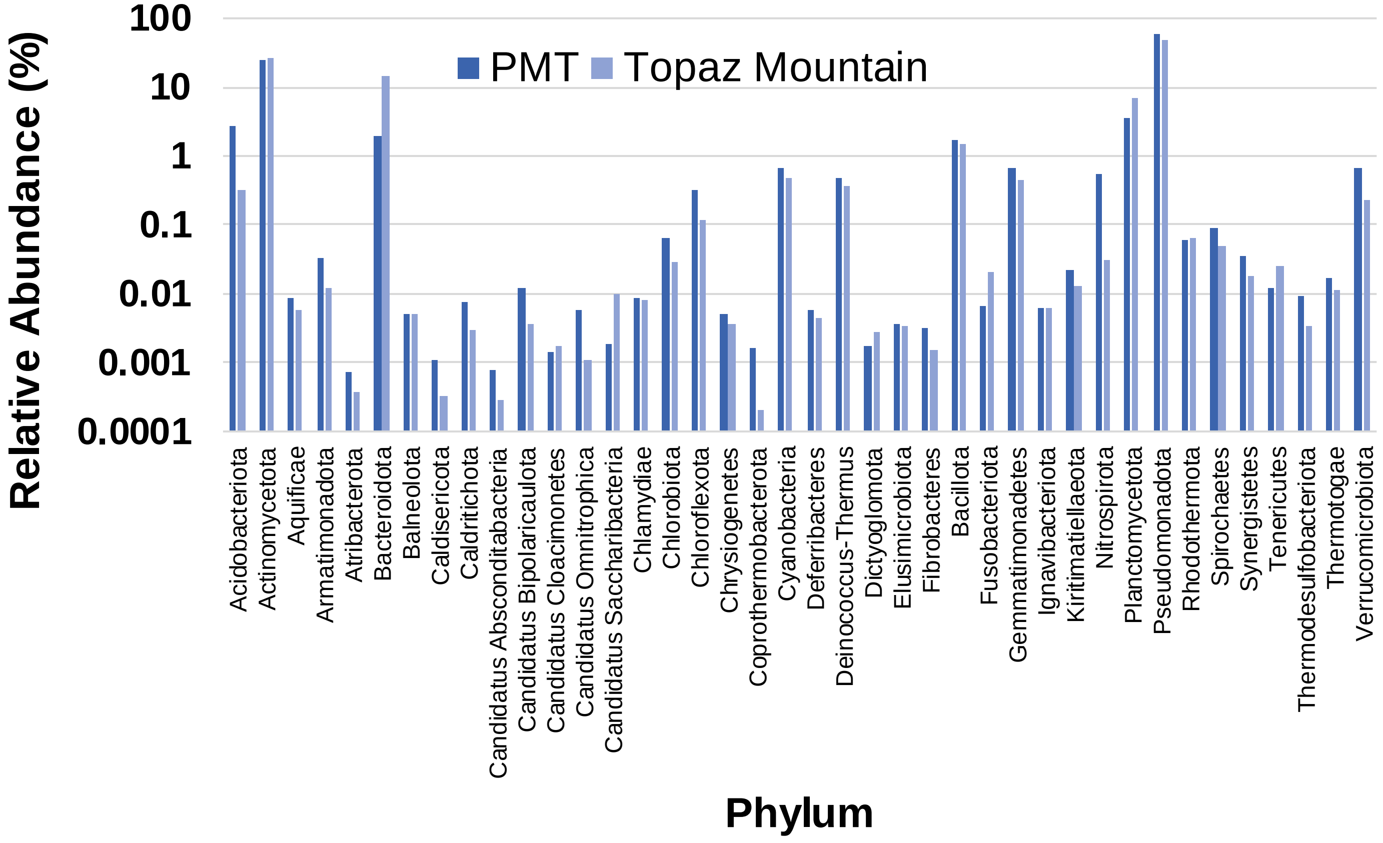

Figure S1. Relative Abundance of Phylum in PMT versus Topaz Mountains. Relative abundance of phyla in PMT (dark blue) and Topaz Mountain (light blue). Note that the axis is on a logarithmic scale.

Supplementary Information

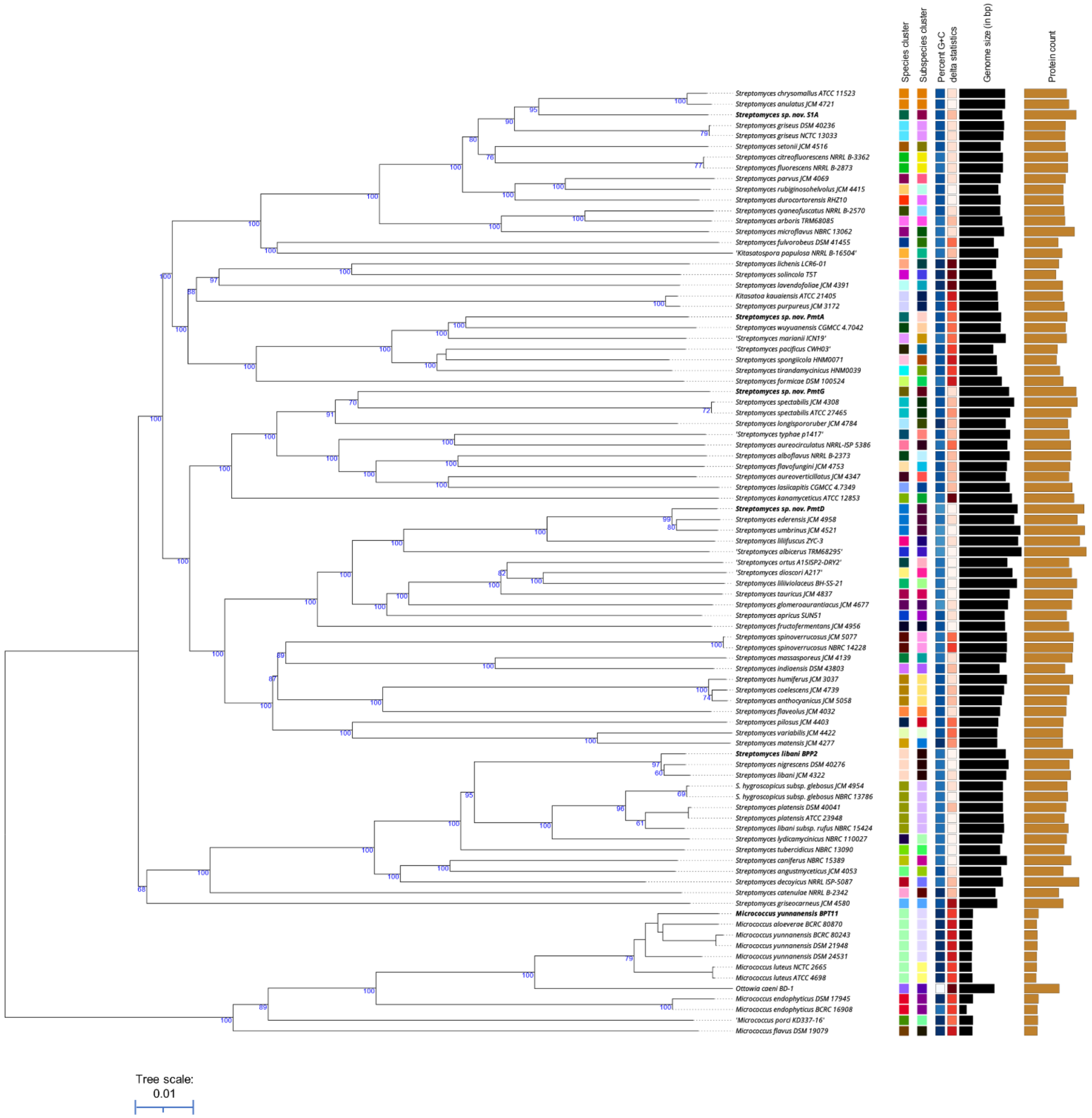

Figure S2. Phylogenomic tree. Taxonomic identification of the six strains sequenced in this study (identified in bold). Within the tree, the species, subspecies, relative GC content, and delta statics of each strain, represented by colored squares, were included. For the species and subspecies, these colors are randomly assigned and simply indicate which branch each one belongs to. For GC content, the darker the blue indicates the higher percentage GC. For the delta statistics, this value demonstrates how closely species are related with lighter colors indicating less related (70% or lower for the digital DNA-DNA hybridization (dDDH) between the genomes from the strains sequenced here and the selected typestrain genomes in most cases). Each strain's relative genome size and relative protein count are shown by black and orange bars, respectively. Bootstrap values for each branch are shown in blue.

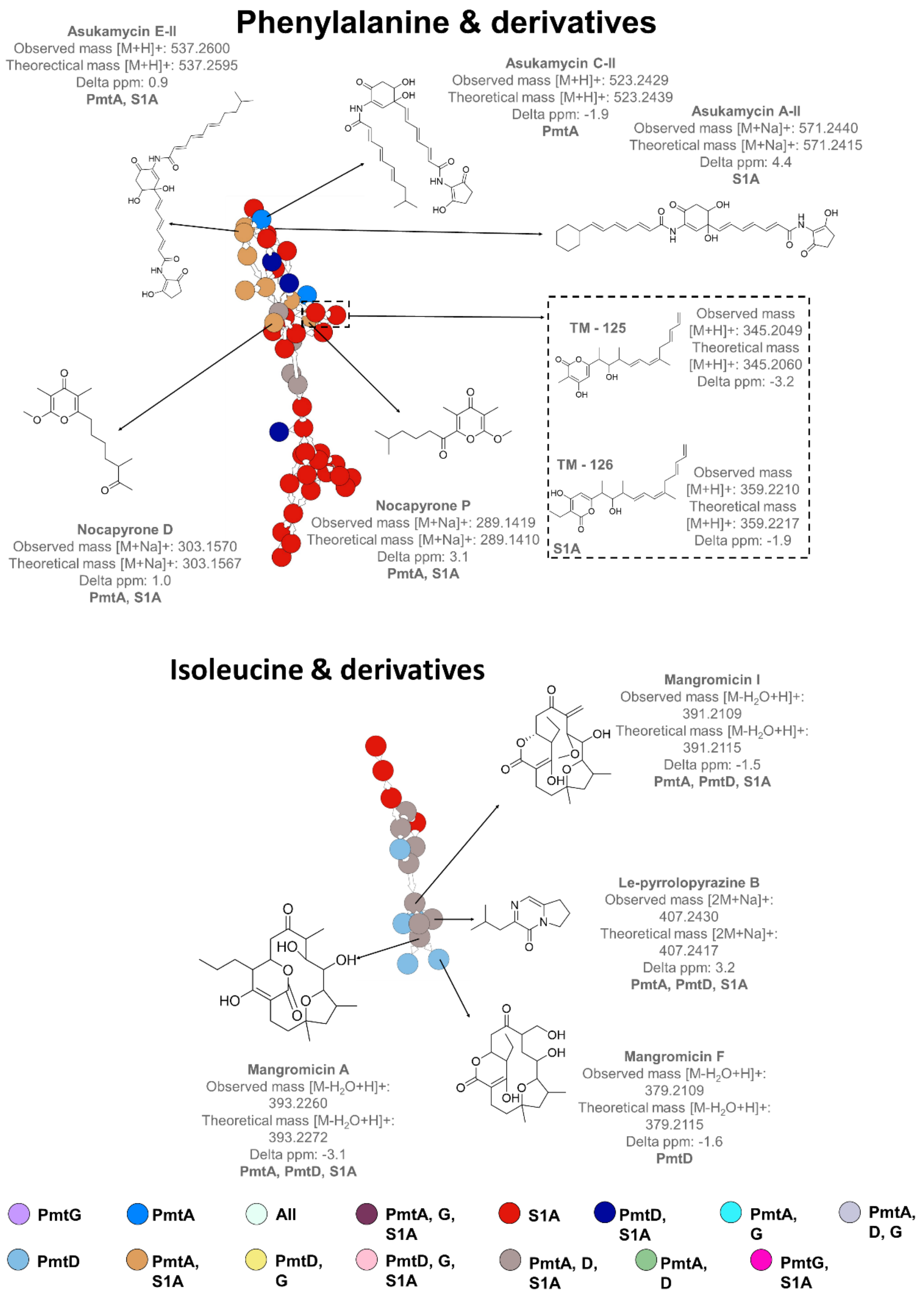

Figure S3. Secondary metabolites predictions for organic acids and derivatives. MolNET-enhanced GNPS networks with potential molecules identified by the SNAP-MS software. Colors indicate the strain of origin.

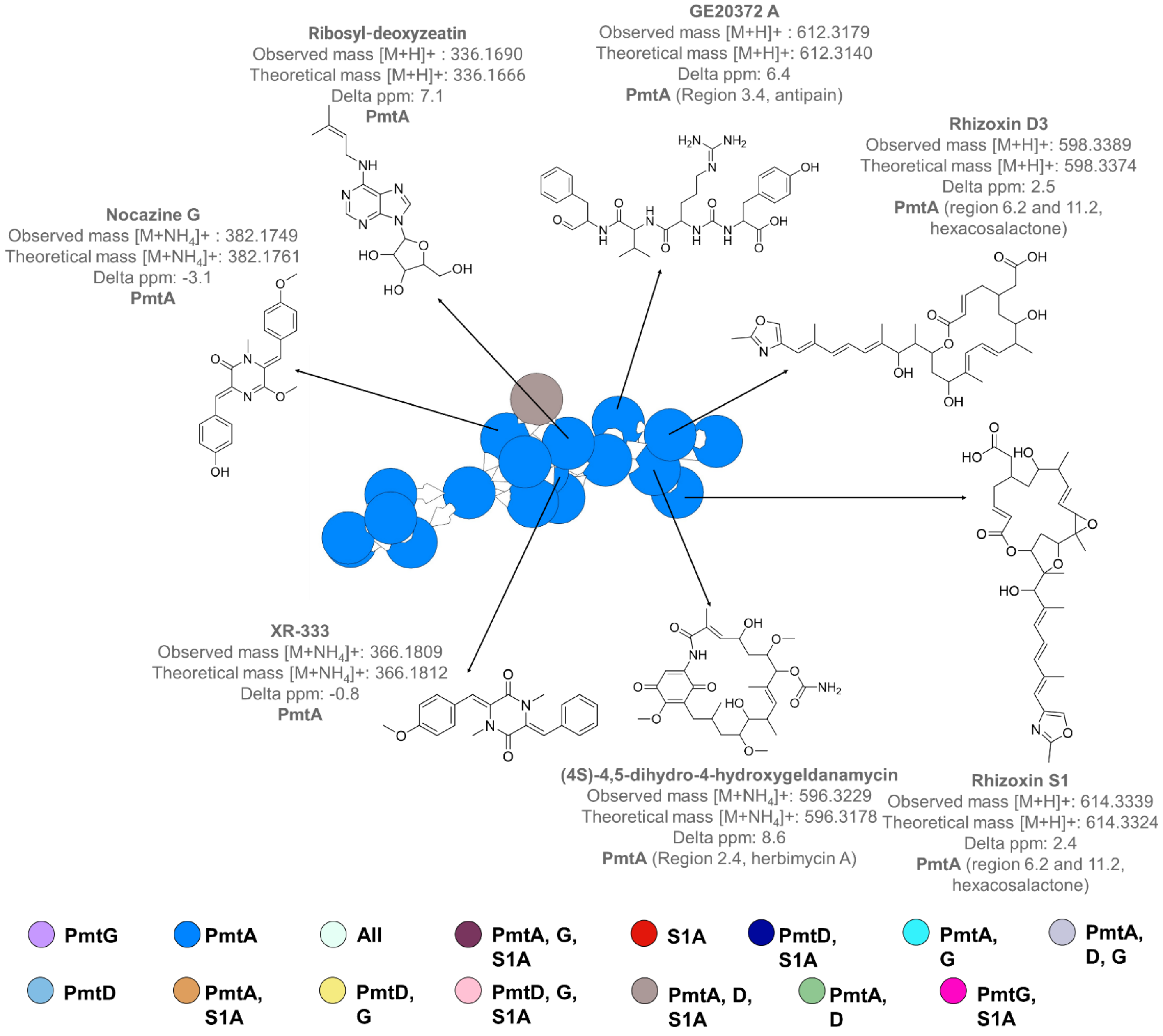

Figure S4. Secondary metabolites predictions for dipeptides. MolNET-enhanced GNPS networks with potential molecules identified by the SNAP-MS software. Colors indicate the strain of origin.

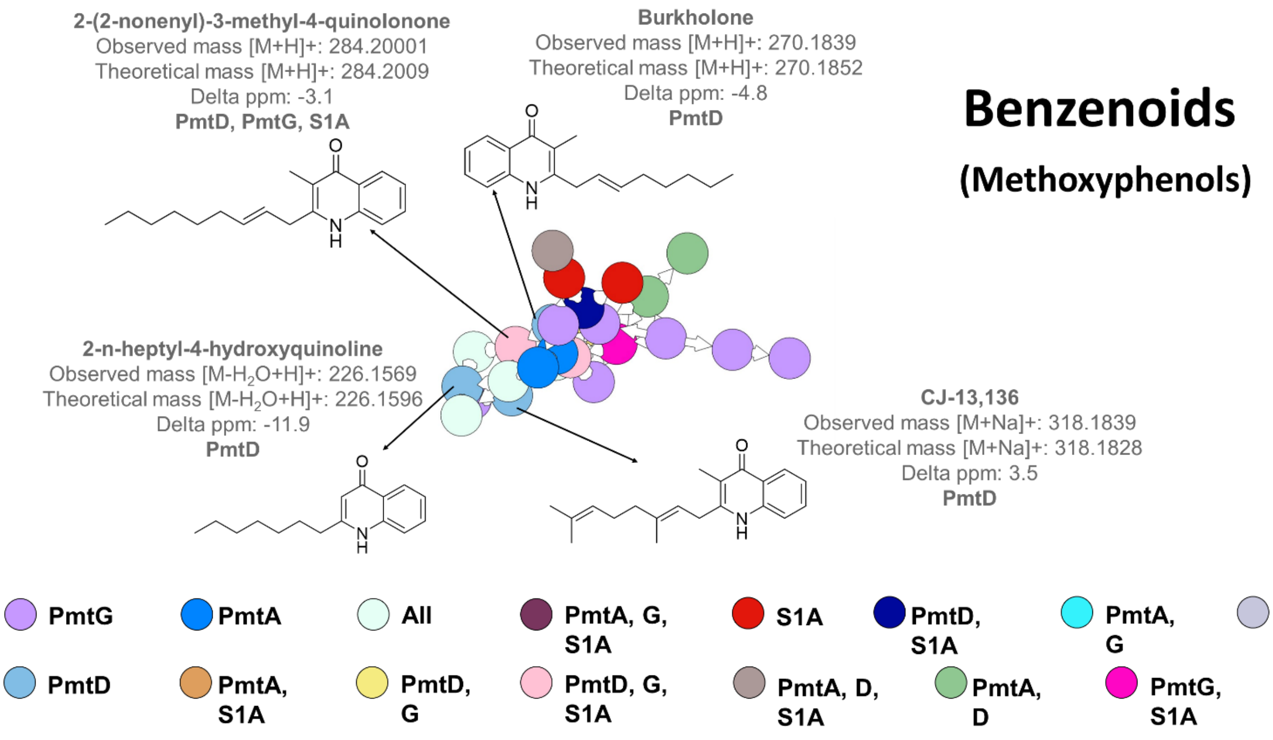

Figure S5. Secondary metabolites predictions for benzenoids. MolNET-enhanced GNPS networks with potential molecules identified by the SNAP-MS software. Colors indicate the strain of origin.

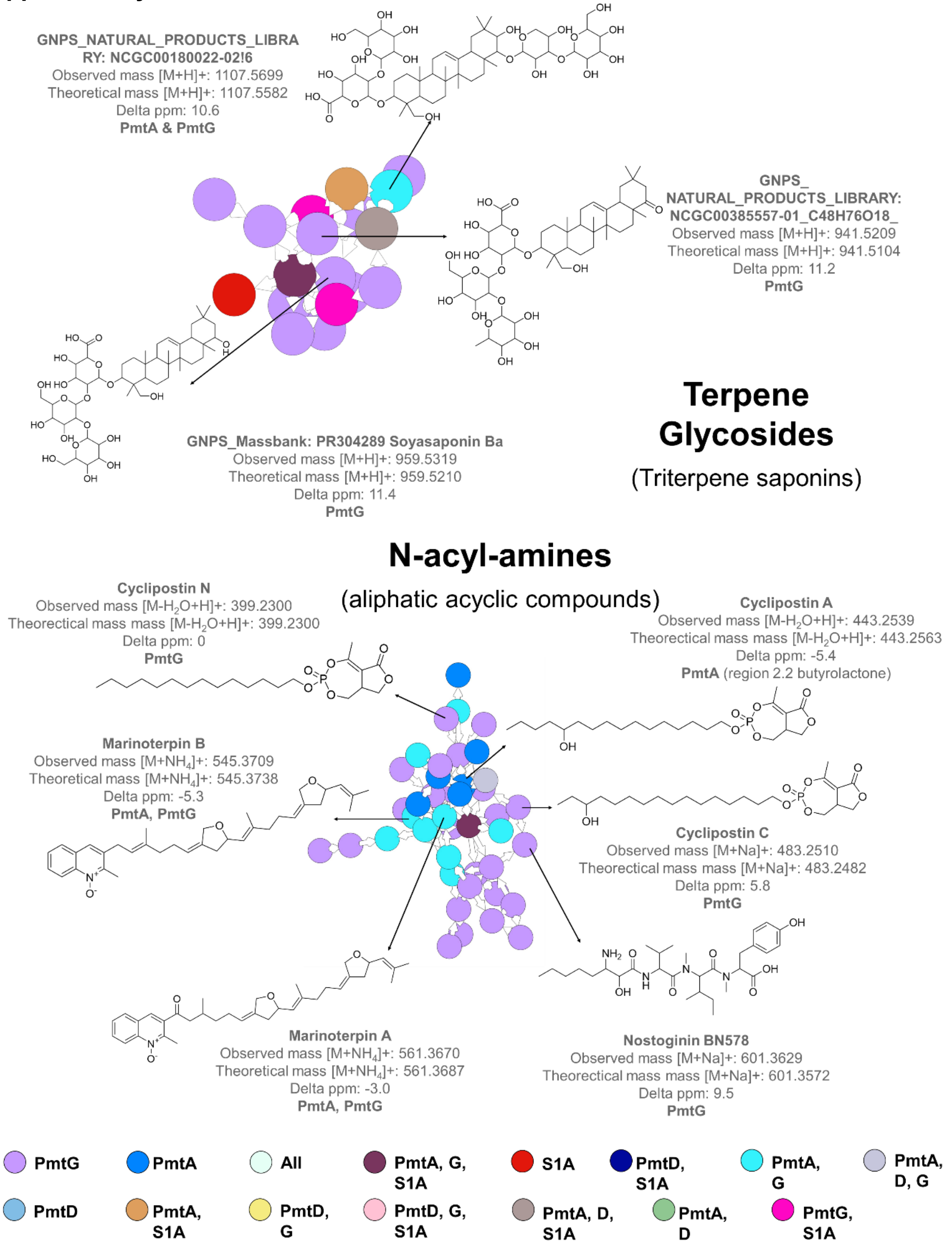

Figure S6. Secondary metabolites predictions for lipid and lipid-like molecules. MolNET-enhanced GNPS networks with potential molecules identified by the SNAP-MS software. Colors indicate the strain of origin.

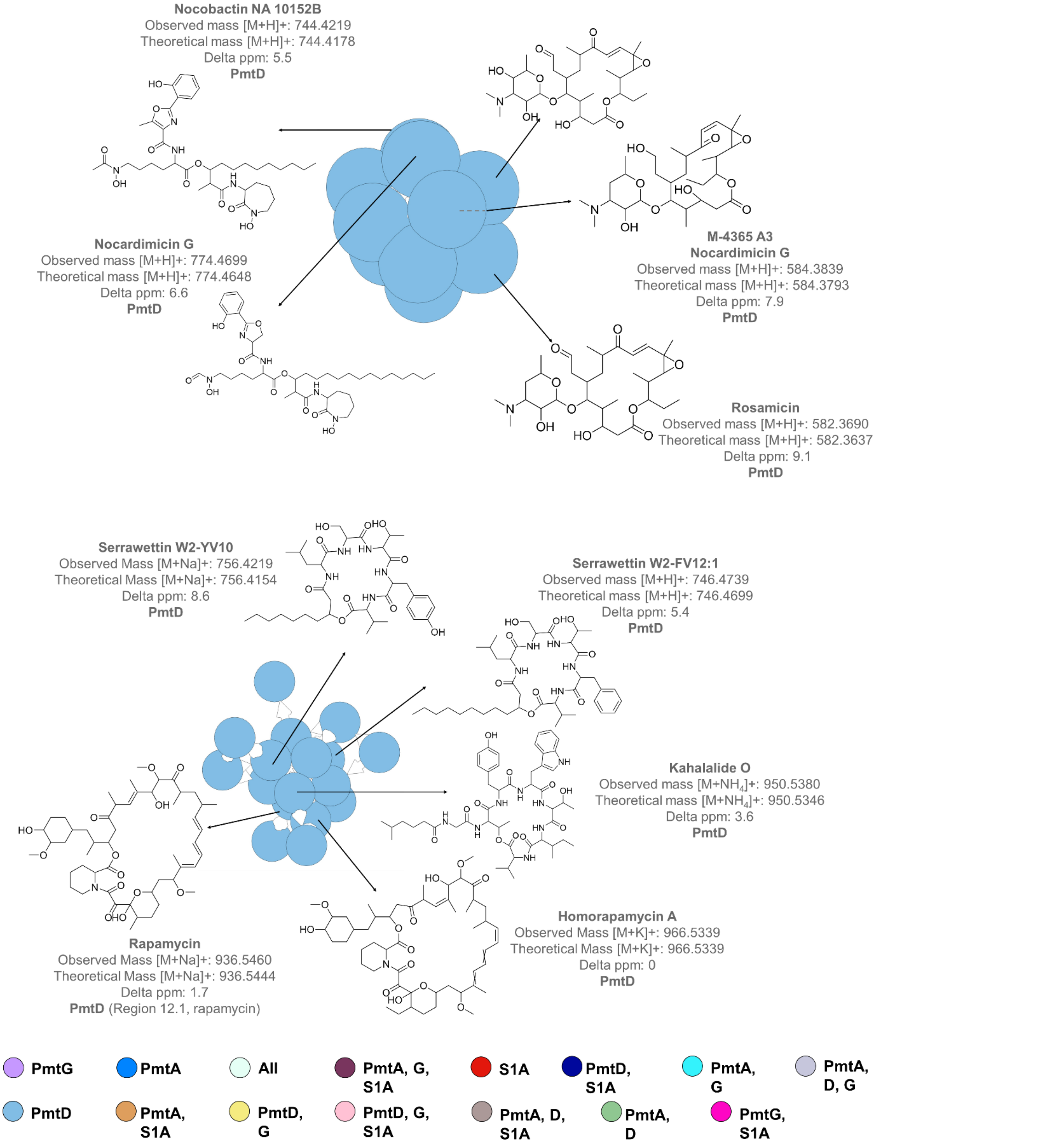

Figure S7. Secondary metabolites predictions for no matches to chemical superfamily, Part 1. MolNET-enhanced GNPS networks with potential molecules identified by the SNAP-MS software. Colors indicate the strain of origin.

No matches

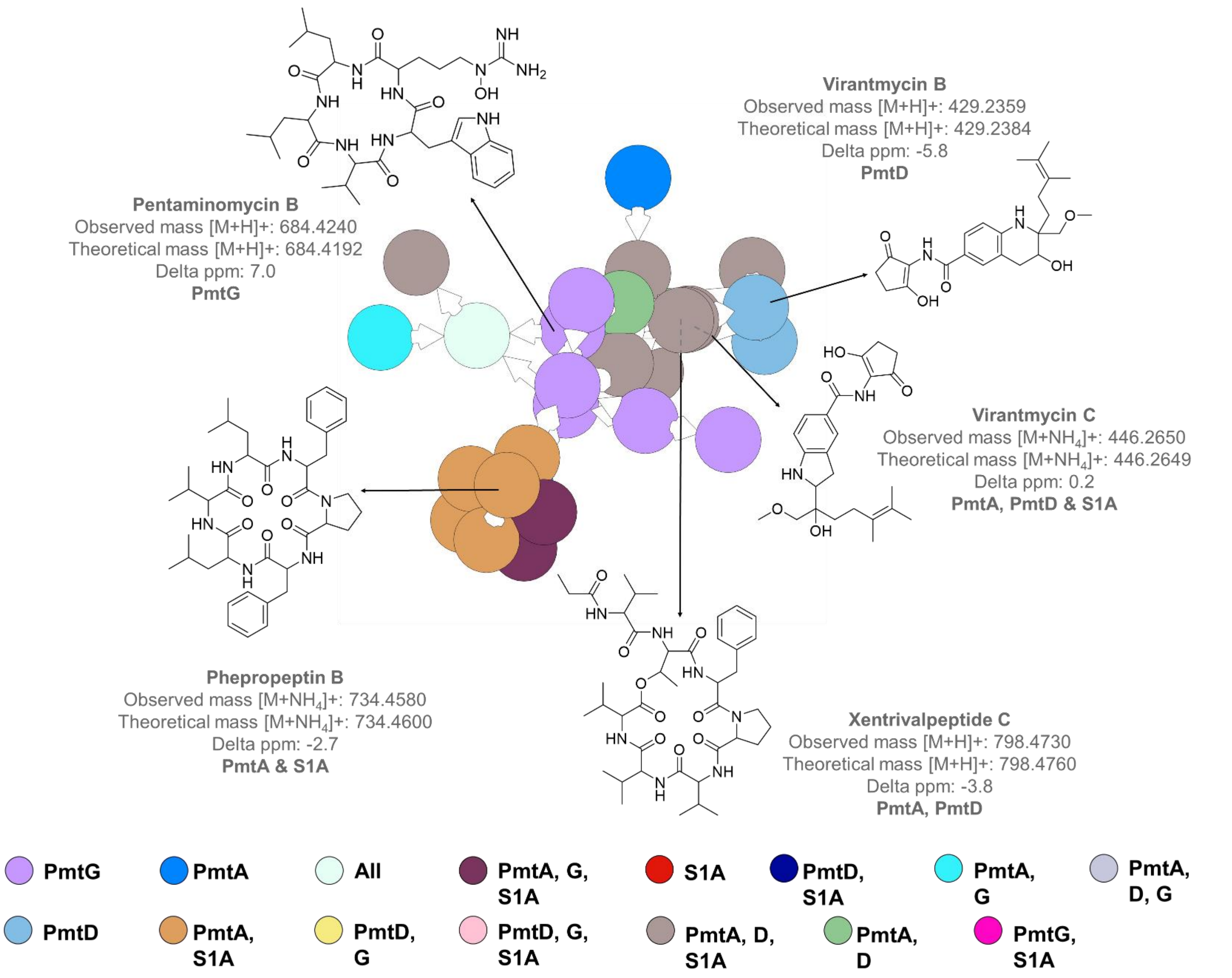

Figure S8. Secondary metabolites predictions for no matches to chemical superfamily, Part 2. MolNET-enhanced GNPS networks with potential molecules identified by the SNAP-MS software. Colors indicate the strain of origin.

Supplementary Information

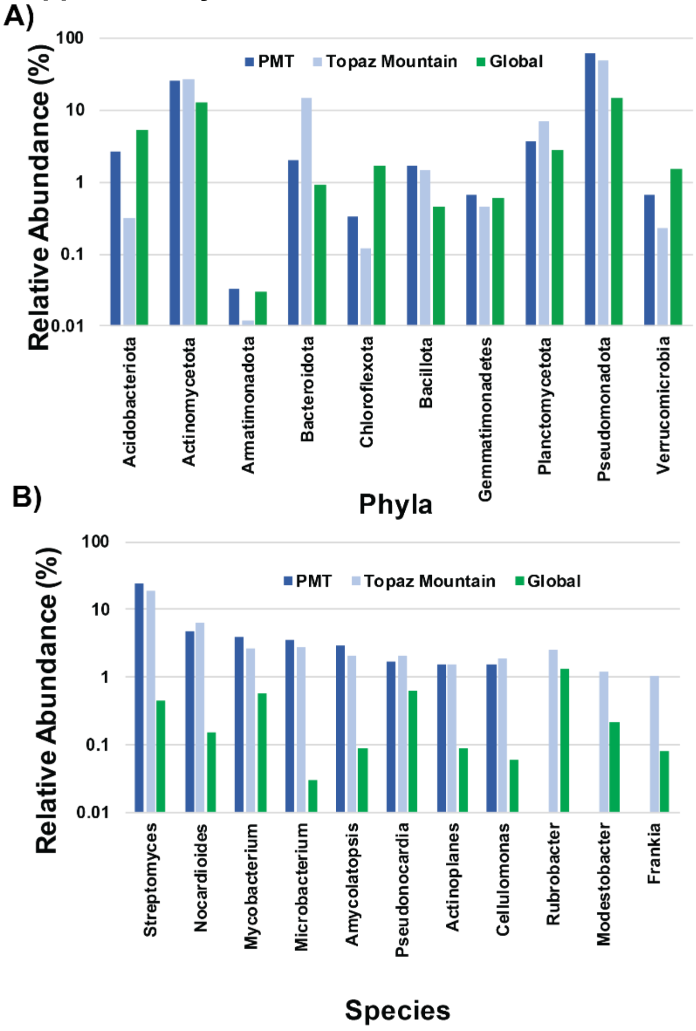

Figure S9. Comparison of observed phyla and genera from global locations to those observed in this study.
A) Top phyla present in approximately half of soils (green, global, data from [1]) along with the levels in the soils evaluated in this study. B) Top genera present in approximately half of soils (green, global data from [1]) along with the soils evaluated in this study. Note: the global study did not have genera data for many of the strains so this may be an underrepresentation.

Supplementary Information
Supplemental References

1. **Delgado-Baquerizo M, Oliverio AM, Brewer TE, Benavent-González A, Eldridge DJ, *et al.*** A global atlas of the dominant bacteria found in soil. *Science (1979)* 2018;359:320–325.
